# The Social Bayesian Brain: Does Mentalizing Make a Difference When We Learn?

**DOI:** 10.1371/journal.pcbi.1003992

**Published:** 2014-12-04

**Authors:** Marie Devaine, Guillaume Hollard, Jean Daunizeau

**Affiliations:** 1 Brain and Spine Institute, Paris, France; 2 INSERM, Paris, France; 3 Maison des Sciences Economiques, Paris, France; 4 CNRS UMR, Paris, France; 5 ETH, Zurich, Switzerland; Duke University, United States of America

## Abstract

When it comes to interpreting others' behaviour, we almost irrepressibly engage in the attribution of mental states (beliefs, emotions…). Such "mentalizing" can become very sophisticated, eventually endowing us with highly adaptive skills such as convincing, teaching or deceiving. Here, sophistication can be captured in terms of the depth of our recursive beliefs, as in "I think that you think that I think…" In this work, we test whether such sophisticated recursive beliefs subtend learning in the context of social interaction. We asked participants to play repeated games against artificial (Bayesian) mentalizing agents, which differ in their sophistication. Critically, we made people believe either that they were playing against each other, or that they were gambling like in a casino. Although both framings are similarly deceiving, participants win against the artificial (sophisticated) mentalizing agents in the social framing of the task, and lose in the non-social framing. Moreover, we find that participants' choice sequences are best explained by sophisticated mentalizing Bayesian learning models only in the social framing. This study is the first demonstration of the added-value of mentalizing on learning in the context of repeated social interactions. Importantly, our results show that we would not be able to decipher intentional behaviour without a priori attributing mental states to others.

## Author Summary

A defining feature of human social cognition is our insight that others' behaviour is driven by their beliefs and preferences, rather than by what is objectively true or good for them. In fact, a great deal of our social interactions are concerned with guessing others' mental states. But is such "mentalizing" of any help for predicting others' behaviour? After all, most animal species seem to cope with this problem without appealing to any form of sophisticated "Theory of Mind". Here, sophistication refers to the depth of recursive beliefs, as in "I think that you think that I think…" Although we are likely to engage in such recursive beliefs whenever our interests are tied up with others' (e.g. in the aim of deceiving them), it is unclear how these beliefs are updated and whether this gives us any advantage when we learn. These are the questions we address in this work, by combining computational and experimental approaches.

## Introduction

What is so special about the way we select the most appropriate action in a social context? We make decisions on the basis of their expected consequences, which we may have to learn from trial and error. However, when this involves predicting other peoples' overt reactions, we almost irrepressibly engage in rich and complex representations of their hidden mental states, such as beliefs, emotions, intentions… In fact, one of the most critical aspects of social inference may be our insight that people's behaviour is driven by their beliefs rather than by physical reality, even if these beliefs happen to be false [1]. In this work, we ask whether this specific aspect of social cognition makes a difference when we learn.

We acquire this insight during early childhood [2], from our developing ability to attribute mental states to others, known as "Theory of Mind" (ToM) or "mentalizing" [3]. ToM is concerned with the interpretation of social signals, from eye gazes and facial expressions to overt behaviour and language, which is why it lies at the core of human social cognition [4]. We know that ToM engages large-scale specific brain networks [5], [6] and that severe neuropsychiatric disorders such as schizophrenia or autism are associated with its impairment [7], [8]. However, current research falls short of an understanding of the computational mechanisms underlying mentalizing, or of a clear demonstration of its added-value for decision making in social exchanges [1]. Here, we take inspiration from recent works in behavioural economics and experimental psychology, which investigate sophisticated mentalizing processes, of the sort that adaptive social skills such as persuading or deceiving proceed from. On the one hand, it has been shown that decisions made in the context of economic games entail recursive thinking of the sort "I think that you think that I think, etc…" [9], [10]. This is essentially because if others' reward depends upon your action, what they believe you will do is relevant for you to predict their behaviour. On the other hand, it has been suggested that simple forms of action understanding conform with Bayesian models of intention recognition [11], [12]. This means that our interpretation of others' actions is optimal, under the insight that others behave according to common sense. Taken together, these ideas yield the "social Bayesian brain" hypothesis, namely: our (Bayesian) brain *a priori* assumes that others are Bayesian too (i.e. others also learn about ourselves) [13]–[15]. In the context of mutual social exchanges, this implies that mentalizing may involve the update of recursive beliefs from the repeated observation of others' overt behaviour. From a modelling perspective, one can define optimal learning rules that are rooted in information theory and are specific to the sophistication of mentalizing agents (i.e., the depth *k* of their recursive beliefs). This is important, because one can now evaluate the added value of some form of mentalizing sophistication, in terms of its ability to decipher intentional behaviour. Critically, our *k-ToM* model predicts that the performance of agents engaged in competitive repeated interactions increases with their ToM sophistication [14].

We test these ideas in the following experiment: we had participants believe either that they were playing a competitive game with each other, or that they were performing a gambling task. In fact, in both conditions, participants were competing against artificial *k-ToM* agents with different ToM sophistication levels. Critically, the task-relevant information (available actions and correct/incorrect feedback), is identical in both framings. Our prediction is twofold: (i) the social framing of the task induces participants to mentalize and thus to engage in recursive inference, and (ii) domain-general learning heuristics that prevail in the non-social framing are vulnerable to artificial mentalizing agents (whose sophistication people cannot grasp). This implies that people should perform better in the social than in the non-social framing of the task, because artificial ToM agents would outsmart learners who do not engage in mentalizing.

## Materials and Methods

### Ethics statement

Our analysis involved de-identified participants' data and was approved by the ethics committee of the Laboratoire d'Economie Expérimentale de Paris (LEEP, Paris Experimental Economics Laboratory). In accordance with the Helsinki declaration, all subjects gave an informed consent.

### Computational modelling

#### 1) *k-ToM* model

In this section, we expose the key steps in the derivation of the *k-ToM* model in the context of repeated two-player games (see also [14]). We used this model both to generate the choices of the participants' (artificial) opponents during the experiment, and in the analysis of participant choices. First, recall that, in its simplest form, a game is defined in terms of a utility table 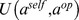, which yields the payoff one gets when making decision 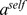 while the other player chooses 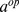. Incentives can be arbitrarily chosen to capture different forms of social exchanges or transactions. In our experiment, we induced social competitive interactions by balancing the gain of the winner by the loss of the loser (“hide and seek” game, cf. Table 1 below).

**Table 1:** "Hide and Seek" utility table as a function of the participant's action 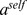 and his opponent's 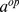.

	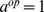	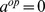
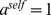	1,0	0,1
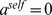	0,1	1,0

In the table entries, the left-hand number if the participant's payoff (the "seeker") and the left-hand number is his opponent's (the "hider").

By convention, actions 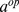 and 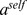 take binary values encoding the first (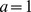) and the second (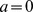) available options. According to Bayesian decision theory, agents aim at maximising expected payoff 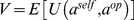, where the expectation is defined in relation to the agent's uncertain predictions about his opponent's next move (see below). Importantly, this implies that the form of the decision policy is the same for all agents, irrespective of their ToM sophistication. In this work, we consider that choices may exhibit small deviations from the optimal decision rule, i.e. we assume agents employ the so-called "softmax" probabilistic policy:
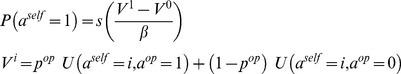
(1) where 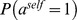 is the probability that the agent chooses the action 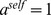, 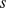 is the sigmoid function and 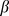 is the exploration temperature that controls the magnitude of behavioural noise. Equation 1 simply says that the probability of choosing the action 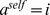 increases with its expected payoff 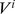. Here, the critical variable is 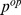: the probability that the opponent will choose the action 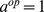.

The repeated observation of his opponent's behaviour 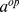 gives the agent the opportunity to learn this prediction. Theory of Mind comes into play when agents consider that the opponent's behavioural tendency 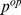 is motivated by his hidden beliefs and desires. More precisely, our "social Bayesian brain" hypothesis implies that ToM agents consider that the opponent is himself a Bayesian agent, whose decision policy 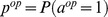 is formally similar to Equation 1. In this situation, one has to track one's opponent's prediction 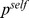 about one's own actions. This makes ToM agents *meta*-Bayesian agents [13], i.e. Bayesian observers of Bayesian agents. In line with [13], this meta-Bayesian inference is recursive ("I think that you think that I think…"). The recursion depth induces distinct ToM sophistication levels, which differ in how they update their subjective prediction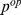.

We define *k-ToM* agents in terms of the way they learn from their opponent's behaviour, starting with *0-ToM*. By convention, a *0-ToM* agent does not attribute mental states to his opponent. More precisely, *0-ToM* agents simply assume that their opponents choose the action 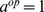 with probability 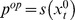, where the log-odds 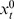 varies across trials 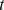 with a certain volatility 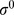 (and 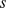 is the sigmoid function). Observing his opponent's choices gives *0-ToM* information about the hidden state 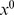, which can be updated trial after trial using the following Bayes-optimal probabilistic scheme:
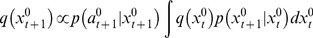
(2)where 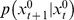 encodes *0-ToM*'s prior belief on the volatility of the log-odds, and 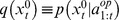 is his posterior belief about the log-odds 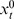 at trial 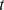, having observed his opponent's behaviour 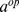 up to trial 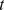. Under these premises, one can derive *0-ToM*'s learning rule, in terms of the change in his prediction about his opponent's next move (we refer the interested reader to Text S1):
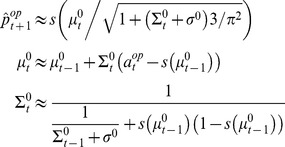
(3)where 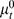 (resp. 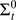) is the approximate mean (resp. variance) of *0-ToM*'s posterior distribution 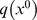. In other words, 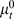 is *0-ToM*'s estimate of the log-odds at trial 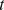, and 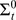 is her subjective uncertainty about it. Inserting 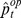 into Equation 1 now yields *0-ToM*'s decision rule. Note that the term 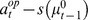 can be thought of as a prediction error, whose impact on learning accounts for changes in the subjective uncertainty 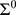. Here, the effective learning rate is controlled by the volatility 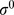. At the limit 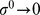, Equation 3 converges towards the (stationary) opponent's choice frequency and Equations 1-3 essentially reproduce "fictitious play" strategies [16], [17].

Equations 1-3 describe how *0-ToM* agents learn and decide, trial by trial. This is the starting point for a *1-ToM* agent, who considers that she is facing a *0-ToM* agent. This means that *1-ToM* has to predict *0-ToM*'s next move, given his beliefs and the choices' payoffs. The issue here is that *0-ToM*'s priors (as well as his exploration temperature) are unknown to *1-ToM* and have to be learned, through their non-trivial effect on *0-ToM'*s choices. More precisely, *1-ToM* agents assume that *0-ToM* chooses the action 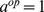 with probability 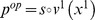, where the hidden states 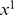 lumps 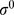 and 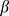 together and the mapping 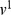 is derived from inserting Equation 2 into Equation 1:
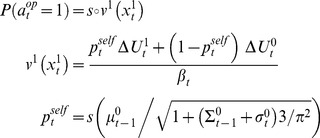
(4)where 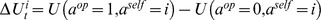 is the net incitation of *1-ToM*'s opponent to pick the first option if *1-ToM* chooses option 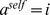. Here, *1-ToM*'s estimate of 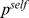 is effectively a second-order belief, i.e. *1-ToM*'s bet about her opponent's prediction about her own next move. Similarly to *0-ToM* agents, *1-ToM* assumes that the hidden states 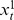 vary across trials with a certain volatility 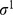, which yields a meta-Bayesian learning rule similar in form to Equation 3 (see Equation 5 below). In brief, *1-ToM* eventually learns how her (*0-ToM*) opponent learns about herself, and acts accordingly.

More generally, *k-ToM* agents (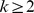) consider that their opponent is a 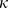 *-ToM* agent with a lower ToM sophistication level (i.e.: 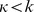). Importantly, the sophistication level 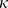 of *k-ToM*'s opponent has to be learned, in addition to the hidden states 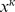 that control the opponent's learning and decision making. The difficulty for a *k-ToM* agent is that she needs to consider different scenarios: each of her opponent's possible sophistication level 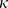 yields a specific probability 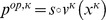 that she will choose action 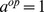.

The ensuing meta-Bayesian learning rule entails updating *k-ToM*'s uncertain belief about her opponent's sophistication level 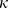 and hidden states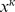:
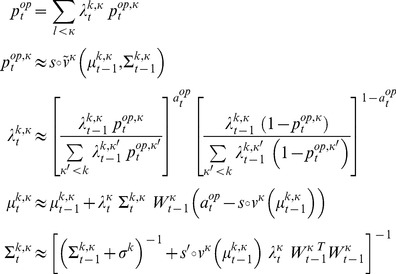
(5)where 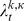
is *k-ToM*'s posterior probability that her opponent is 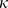 *-ToM*, and 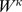 is the gradient of 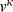 with respect to the hidden states 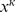. Here, the mapping 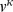 is obtained by the recursive insertion of Equation 5 into Equation 1 (as in Equation 4), and 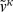 is defined implicitly in terms of the expectation operator, as follows: 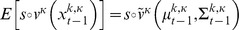. Equation 5 is but a compact formulation of how the summary statistics (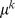, 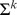 and 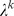) of *k-ToM*'s posterior distribution 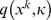 evolve from trial to trial. Both Equations 3 and 5 have been derived using a variational approach to approximate Bayesian inference [18]–[20]. We refer the interested reader to a previous theoretical paper [14]. Although Equation 5 is slightly more complex than Equation 3, note that learning is still driven by a simple prediction error term. However, there is an interaction between the beliefs on the opponent's sophistication level and hidden states. For example, one can see that 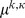 and 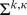 are left unchanged if the 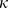-ToM scenario is unlikely, i.e. if 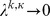. Also, 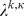 increases in proportion to how likely was the opponent's last choice under the 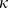 *-ToM* scenario 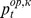, which depends upon 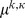 and 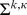.

Finally, note that *k-ToM* models do not differ in terms of the number of their free parameters. More precisely, *k-ToM*'s learning and decision rules are entirely specified by their prior volatility 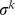 (cf. Equations 3 and 5) and behavioural temperature 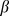 (Equation 1).

This concludes the mathematical exposition of our meta-bayesian model of ToM agents.

**Figure 1:**
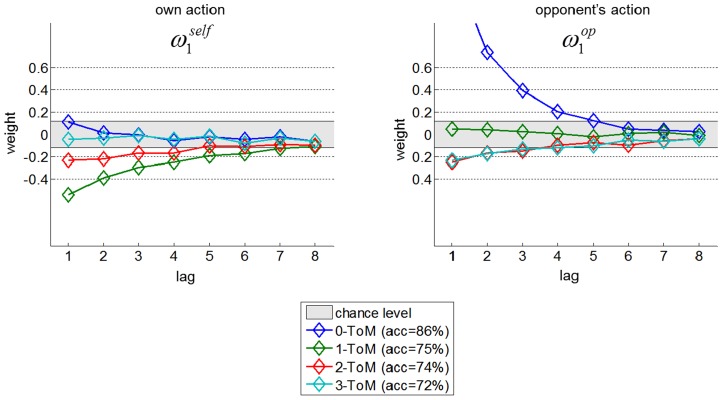
Volterra decomposition of *k-ToM*'s response. Left: impulse response to *k-ToM*'s own action (x-axis: lag 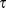, y-axis: Volterra weight 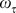). Right: impulse response to *k-ToM*'s opponent's action. ToM sophistication levels are colour-coded (blue: *0-ToM*, green: *1-ToM*, red: *2-ToM*, magenta: *3-ToM*). The grey shaded area denotes chance level.

At this point, one may not have a clear intuition about how such *k-ToM* agents react to their opponents' choices. We thus performed Volterra decompositions of simulated choice sequences of artificial *k-ToM* agents playing "hide and seek" against a random opponent. In our context, this means regressing *k-ToM*'s simulated choices onto (i) her opponent's past choices, and (ii) her own past choices (see Text S1). In brief, a positive Volterra weight captures a tendency to reproduce or copy the corresponding action. Fig. 1 shows the estimated Volterra kernels of *k-ToM* agents, averaged across a thousand Monte-Carlo simulations. Chance level was derived as the extremum Volterra weights estimated for a random choice sequence. We also evaluate Volterra's fit accuracy, in terms of the percentage of correct choice predictions.

One can see that *0-ToM* has a strong tendency to imitate the behaviour of her opponent (positive Volterra weights 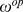 of opponent's actions). In contradistinction, *1-ToM* anticipates this and thus tends to alternate her own choices (negative Volterra weights 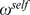 of own actions). *2-ToM* depicts a pattern that mixes the anticipation of *1-ToM* (picking his opponent's unchosen action) and *0-ToM* (alternating his own choices). Finally, we note that Volterra's fit accuracy decreases with ToM sophistication (from 86% to 72%). This is because nonlinearities in the behaviour of *k-ToM* agents (as induced by, e.g., changes in their belief about their opponent's sophistication) cannot be completely captured without higher-order Volterra kernels.

#### 2) Other agents' models

The above *k*-ToM model was used both in the experimental paradigm (artificial players), and in the statistical data analysis (participants' behaviour). In order to test our social Bayesian brain hypothesis, we need to compare our *k-ToM* model with other non-Bayesian and/or non-mentalizing models of peoples' choice sequences. Table 2 below summarizes the characteristics of the models we included in the comparison set. As can be seen, the comparison set can be partitioned into either Bayesian (B+) versus non-Bayesian (B-) model families, or ToM (T+) versus no-ToM (T-) model families. We will use this factorial structure of the comparison set when performing group-level Bayesian model selection. Let us now briefly describe the rationale behind these agent's models:

**Table 2:** Summary of the models included in the comparison set.

Model's name	Bayesian	mentalizing	number of free parameters
*k-ToM* ( 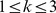 )	yes (B+)	yes (T+)	3
*0-ToM*	yes (B+)	no (T−)	3
*HGF*	yes (B+)	no (T−)	5
*n-BSL* ( 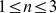 )	yes (B+)	no (T−)	3
*k-Inf* ( 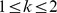 )	no (B−)	yes (T+)	3 (*1-Inf*), 4 (*2-Inf*)
*RL*	no (B−)	no (T−)	3
*WSLS*	no (B−)	no (T−)	2
*Nash*	no (B−)	no (T−)	1

For all agent's models (including *k-ToM*), the probability of choosing the action 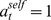 at trial 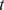 can be written using the softmax policy of Equation 1, augmented with an unknown bias term. This formulation is convenient because models only differ in terms of the underlying dynamics of hidden states that determine either the agent's prediction about their opponent's next move 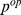 (as in, e.g., Equation 3) or directly options values 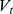 (see below):

- *hBL* (hierarchical Bayesian Learner): this model is a hierarchical extension of *0-ToM*, which includes a Bayesian update rule for the volatility 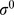 of the opponent's log-odds. This yields a sophisticated non-mentalizing agent that can adapt its learning rate over the course of the experiment. Augmenting *0-ToM* with such a learning rule essentially cost two additional parameters that control the coupling between the volatility and the log-odds. We refer the interested reader to [21].
- *n-BSL* (Bayesian Sequence Learner): this is another extension of *0-ToM*, which optimally tracks the frequency of the opponent's choice sequences of length *n*. More precisely, *n-BSL*'s prediction about her opponent's next move 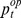 depends upon the previous *n* actions, i.e.: 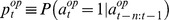. Although the number of her belief's sufficient statistics increases exponentially with n (there are 2^n^ sequences of length n), *n-BSL*'s corresponding update rules are simple duplicates of Equation 2.
- *k-Inf* (“Influence” model): this is a non-Bayesian mentalizing agent that can be regarded as an analogous to *k-ToM*, in that she accounts for how her own actions influence her opponent's strategy. For example, *1-Inf* uses the following heuristic tracking rule of her opponent's actions [22]:

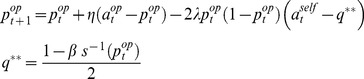
(6)where 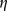 (resp. 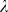) controls the relative weight of *1-Inf*'s prediction error (resp. the “influence” correction term). Note that the “influence” correction term is proportional to *1-Inf*'s estimate of her opponent's prediction error. Equation 4 can be augmented with a second-order correction term, which incorporates the knowledge that the opponent is itself using an influence model. This yields *2-Inf*'s update rule:
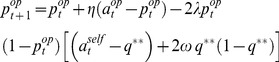
(7)
  where 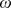 now controls the weight of *2-Inf*'s opponent's (first-order) influence correction term. Note that we did not consider higher order correction terms.

- *RL* (reinforcement learning): at each trial, the agents update the value of the chosen option in proportion to the reward prediction error [23]

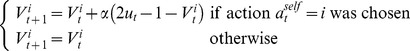
(8)where 
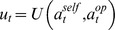
 is the last game outcome and 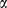 is the (unknown) learning rate.

- *WSLS* (win-stay/lose-switch): at each trial, the agent repeats her last choice if it was successful and alternates otherwise [24]:

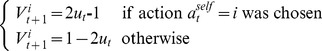
(9)

- *Nash:* this is a probabilistic policy that prevents the other player from controlling his expected earnings. In "hide and seek", the (static) Nash strategy consists in choosing any of the two options with a fixed probability of 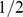. This can be done by fixing the options values as follows: 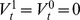. Note that here, the Nash policy is strictly equivalent to a random chance model (with a potential bias towards one of the alternative options).

### Experimental methods

#### 1) Participants

In total, n = 29 subjects (15 females, mean age  = 22.5, SD  = 3.8) without history of neurological or psychiatric disease were recruited via e-mail within an academic database. Three participants were excluded from the analysis due to very low performance in a 3-back control task (see below). Participants were paid a minimum of 5 € plus an additional monetary bonus that depended upon their performance in the different tasks (see Text S1). They were instructed about monetary earnings prior to the experimental session.

#### 2) Main task

In our main experiment, participants performed four games of “hide and seek” and four sessions of the Casino gambling task. Our rationale for choosing the "hide and seek" game is twofold: (i) one can show that, in this game (as opposed to, e.g., cooperative games), expected performance increases with ToM sophistication [14], (ii) it lends itself easily to a non-social framing. Participants were divided in two subgroups, each of which performed the experiment at the same time in the same room. This was required to make them believe that they were playing against each other (social framing). Since LEEP policy prevents deceiving instructions, participants were not explicitly told they were playing against each other. Instead, in the social condition, participants were instructed that they would “play hide and seek against four different players” and that each of them would “be attributed one of the two possible roles: hider or seeker”. The respective role of seekers and hiders were explained before the beginning of the experiment. Eventually, all participants were privately informed (through instructions on their computer screen) that they were “seekers”. In the non-social framing, participants were instructed they would "perform four sessions of repeated choices between two slot machines" and that "only one slot machine would be winning on any given trial". In both conditions, participants were given feedback (correct/incorrect) on their choice at each trial. Strictly speaking, in both framings, participants were not given any information regarding the true feedback mechanism, apart from the fact that there was a unique correct option at each trial (i.e. they knew the counterfactual outcome: if one option led to “success” the other one necessarily led to “fail”).

**Figure 2:**
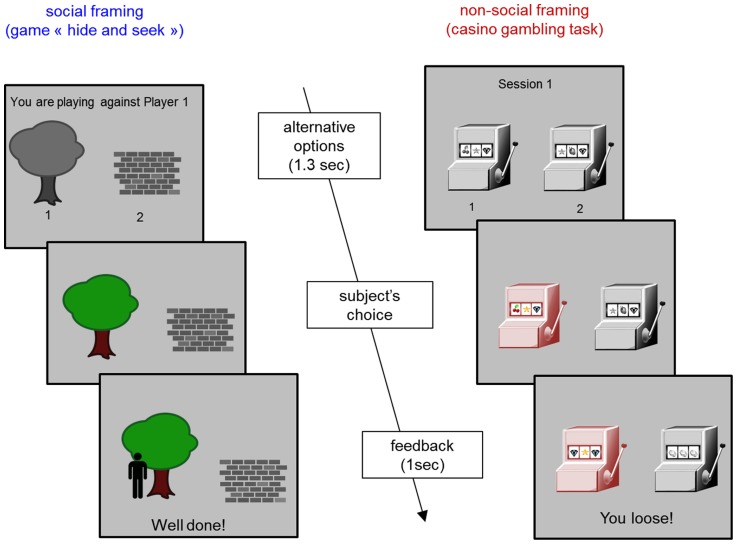
Main task's experimental paradigm. Left: social framing ("hide and seek" game). Right: non-social framing (Casino game). At each trial, participants have 1300 msec to pick one of the two options (social framing: wall or tree, non-social framing: left or right slot machine). Feedback is displayed for 1 sec, for both framings this feedback includes if the subject won or lost and the actual winning option by showing a character picture (social framing) or three identical coins (non-social framing).

In fact, each game/session was played against a specific algorithm (2×4 factorial design, cf. Fig. 2), namely: a random sequence with a 65% bias for one option (bias was counterbalanced between the two framings within participants), a *0-ToM* agent, a *1-ToM* agent and a *2-ToM* agent. Critically, *0-ToM*, *1-ToM* and *2-ToM* algorithms are all learning agents (i.e. they adapt to the participant's choices), but only *1-ToM* and *2-ToM* engaged in (artificial) mentalizing. Note that the random biased opponent (*RB*) serves as a control condition for non-specific motivational or attentional confounds on the performance difference between the two framings (e.g., people being more willing to engage in a game with other human players). The order of opponents was randomized for each participant.

Each game/session included sixty trials in which participants had to choose between two options (two hiding places or two slot machines) in less than 1300 msec. If they were too slow, the opponent's choice was not revealed (about 0.5% of trials) and the point was attributed to the other player. Feedback was then revealed for 1 sec after which a new trial began and the total number of correct trials was given at the end of each session. Before obtaining their final earnings, participants had to fill in a debriefing form, in which they could describe verbally their impressions and strategies. Note that participants expressed no suspicion regarding the task framing.

#### 3) Secondary tasks

In addition to the main task, participants performed three tasks assessing executive functions. First, behavioural flexibility was assessed through the number of perseverative responses in a modified card sorting task [25]. Second, inhibitory control was measured as the sensitivity index *d′* in a Go/No Go task [26]. Finally, working memory capacity was measured as the sensitivity index *d′* in a 3-back task [27], [28].

In addition, participants completed the Empathy Quotient test [29]. For completeness, they were also asked to perform three standard ToM tasks. First, their aptitude to acknowledge the difference between their own and others' beliefs was measured as the average probability rating attributed to the correct response in “Vicky's Violin” false belief task [30], [31]. Second, their ability to reason about embedded narratives was measured by the ratio of correct answers (mental states vs control) in the “Imposing Memory task” [32]. Finally, their accuracy in discriminating between distinct intentional and emotional states was scored using the categorization accuracy in the Frith-Happé animation task [33].

Participants performed all the above tasks in the following order: “hide and seek”, “Vicky's Violin” task, the modified card sorting task, the Casino gambling game, the Frith-Happé animation task, the Go-No Go task, the empathy quotient, the 3-back task, Vicky's Violin task (2) and finally, the imposing memory task. In total, the experiment lasted roughly one hour and a half.

### Statistical data analyses

All statistical data analyses (including ANOVAs) were performed using the VBA toolbox (http://code.google.com/p/mbb-vb-toolbox/) [34]. Note: although we report summary statistics that are not corrected for multiple comparisons, we indicate the family-wise error rate threshold (*FWER_5%_*) when necessary.

#### 1) Behavioural performance

First of all, we performed a design sanity check, i.e. we verified that there was no difference in opponents' biases across framing conditions (cf. Figure 1 in Text S1). Testing our main hypothesis thus reduces to asking whether participants perform significantly better in the social than in the non-social framing. Here, peoples' performance or earning is defined as the difference between the numbers of correct and incorrect trials, i.e.: 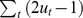, where the game outcome 
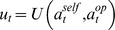
 at any given trial 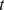 is either “correct” (1) or "incorrect" (0). Under the null (*H_0_*), one is as likely to be correct as incorrect, i.e.: 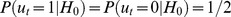. It follows that one can derive the probability distribution 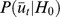 of average cumulative earnings 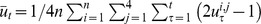 as a function of trial index, where 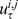 is the game outcome at trial 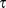 for participant 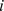 against opponent 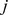. We used this to identify the classical 5% false positive rate threshold, i.e. the critical average earning 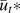 that yields 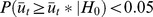. Classical significance testing of observed performance in the main task thus reduces to a direct comparison with 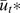, which we did for earnings in both framing conditions, as well as for the difference in earnings between framings.

Further, we assessed the effect of framing, of opponent and their interaction using a pooled-variance ANOVA on final earnings. For the sake of simplicity, we modelled the opponent's factor in terms of the linear effect of sophistication onto performance. In addition, we also performed tests of condition-specific effects. Since the latter did not correspond to *a priori* hypotheses, we indicated the corrected thresholds for completeness.

Finally, we analysed the impact of executive functions, empathy or (secondary) ToM tasks onto peoples' performance in each framing condition of the main task using a general linear model, which also included participants' age and gender. More precisely, we used framing-specific omnibus F-tests to test for any effect of performances in the seven secondary tasks on peoples' final earnings (averaged across opponents). We also performed the same analysis on the difference in performance (between framings).

#### 2) Volterra decompositions of choice sequences

Volterra series allow a systematic decomposition of dynamical systems' input-output relationships, where the output is typically a function of the history of past inputs. In our context, we assume that each choice results from the (logistic) convolution of both players' past actions. This means that Volterra decompositions reduce to estimating the impulse response to one's own and opponent's actions, respectively (see Text S1 for more details).

We performed Volterra decompositions of each participant's choice sequence, in each condition of the main task. We then assessed the effect of framing, of opponent and their interaction using a pooled-variance ANOVA on each Volterra weight separately. In addition, Volterra decompositions of artificial *k-ToM* agents (cf. Fig. 1) serve as a reference point for interpreting participants' Volterra kernels. More precisely, they define "best *k-ToM* responses" to each opponent type (for instance, *1-ToM* is a "best *k-ToM* response" to *0-ToM* since she holds a correct model of her opponent), which one can compare each participant's response to. In particular, the similarity to the "best *k-ToM* response" is a proxy for the optimality of people's learning rule when playing against ToM agents.

#### 3) Bayesian model comparison

In total, we included thirteen agent models (see Table 2) and the Volterra decomposition (for reference) in the statistical comparison. All these models were augmented with a potential (session-specific) bias towards any of the two options, which was included in the logistic likelihood function (cf. Equation 1). Note that these models differed in the number of unknown parameters, which ranges from 2 parameters for *WSLS*, to 17 for *Volterra*. Since these were allowed to vary between subjects (and, within subjects, across conditions), one has to account for model complexity when evaluating how likely these models are given the participants' choice sequences. This was done by evaluating the marginal likelihood or Bayesian model evidence, under a variational Laplace approximation [20]. Eventually, we obtained 14×26×2×4 = 2912 model evidences (14 models, 26 participants, 2 task framings, 4 opponents). These were then inserted into a group-level random-effect Bayesian model comparison (RFX-BMS) [35]. This analysis treats models as random effects that could differ between subjects, with an unknown population distribution (described in terms of model frequencies/proportions). This is particularly useful in our context, because we assume that different individuals may have distinct ToM sophistication levels. In every analysis we report the exceedance probability (EP) associated with models (or family of models), which corresponds to the posterior probability that a given model is the most frequent one in the population. Relevant methodological details of RFX-BMS are summarized in Text S1. First, between-condition comparisons allowed us to ask whether models were the same across task conditions [36]. It confirmed that, in contrast to the opponent factor, the task framing is likely to induce differences in model attributions. We then summed log-evidences over opponents (fixed effect across opponents), and performed framing-specific RFX-BMS. This allowed us to estimate model frequencies and ToM/no-ToM family exceedance probabilities for both task framings. The proportion of ToM sophistication levels was derived by re-performing an RFX-BMS, having restricted the set of models to the winning family.

## Results

### Behavioural performance results

**Figure 3:**
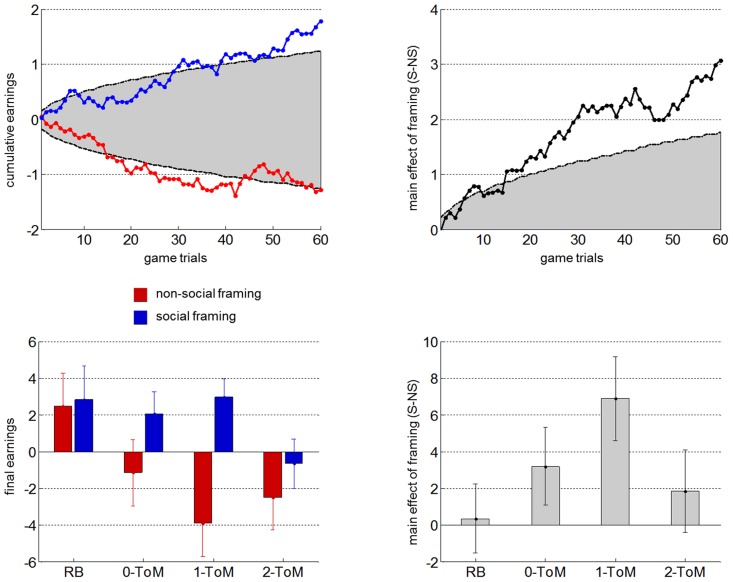
Group-level performance results. Top-left: average cumulative earnings *ū_t_* (y-axis) in the social (blue) and non-social (red) framings, as a function of trials *t* in the game (x-axis), overlaid on the chance 5% false positive rate threshold (grey shaded area). Top-right: average difference in cumulative earnings *ū_t_* (social minus non-social) as a function of trials *t* in the game, overlaid on the chance 5% false positive rate threshold. Bottom-left: group average cumulated earnings against the four different opponents (red: non-social framing, blue: social framing). Errorbars depict one standard error. Bottom-right: group average difference (social minus non-social) in cumulated earnings against the four different opponents.

Fig. 3 summarizes the group results on the behavioural performance in the main task. Overall, the pattern of mean performances follows our predictions.

Let us first consider the top-left panel of Fig. 3, which depicts the dynamics of the group mean cumulative earnings (averaged across opponents) for both framing conditions, overlaid on the chance 5% false positive rate threshold. One can see how the effect size unfolds over time. In particular, it is reassuring to see that participants' performance tends to reach statistical significance almost from the start of the experiment onwards. When summarizing the performance in terms of final earnings: people significantly win in the social framing (*ū_60_* = 1.79, p = 0.008), whereas they significantly lose in the non-social framing (*ū_60_* = −1.28, p = 0.047) despite positive earnings against *RB* in the non-social condition (cf. Fig. 3, bottom-left panel). The framing effect is even clearer on the top-right panel of Fig. 3, which depicts the dynamics of the difference in average cumulative earnings between framings. In brief, the framing effect becomes significant at about trial t = 15, and increases in size as time unfolds (to reach *ū_60_* = 3.07, p = 0.002 at the end of the game). We refer the interested reader to Figure 2 in Text S1 for further information regarding the dynamics of condition-specific earnings.

Now, as one can see on the bottom-left panel of Fig. 3, participants' final earnings seem to depend upon both the framing and the opponent type. More precisely, in the social framing, participants seem to win against all artificial agents except *2-ToM* (null earnings). In contrary, in the non-social framing, participants seem to lose against all mentalizing opponents, be even with *0-ToM*, and win against *RB*. This view is largely consistent with results of the ANOVA on peoples' final earnings: In addition to the main effect of framing (F = 7.49, p = 0.007), participants' performance significantly decreases with the sophistication of their opponent (F = 6.96, p = 0.009), but show no interaction of framing and opponent (F = 0.89, p = 0.35). Including participants' performance in the seven secondary tasks (as well as their age and gender) as confounding factors in the ANOVA did not change these results.

When looking more closely at condition-specific effects (cf. Fig.3 bottom-right panel), we found that the opponent, against which participants' performance showed the strongest framing effect was *1-ToM* (t = 2.9, p = 0.003; FWER_5%_  = 0.0032). This makes sense, if we assume that peoples' effective ToM sophistication is higher (resp. lower) than 1-ToM in the social (resp., non-social) framing. Note that the mean performance in the control condition (*RB*) shows no difference between the social and non-social framings (t = 0.1, p = 0.43). This is important, because it implies that the difference in mean performance against *1-ToM* is unlikely to be due to motivational or attentional confounds (which would also induce differences against *RB*).

At this point, we looked at inter-individual differences to strengthen our results' interpretation. First, we asked whether any inter-individual variability in peoples' performance could be explained by inter-individual differences in the seven secondary cognitive tasks. Interestingly, we found no significant effect on peoples' performance in the main task, irrespective of the task framing or the opponent's sophistication (see Text S1 for further details). This is important, because this implies that peoples' capability to outsmart artificial mentalizing opponents is not influenced by executive functions or empathy. Next, we asked whether idiosyncratic differences in motivational and/or attentional states could drive the inter-individual variability in our main task. We reasoned that if this was indeed the case, people who win more than others in the social framing should also win more in the non-social framing. We thus focused on the correlation between peoples' performance in the social and in the non-social framings. To begin with, we found no correlation between average performances in the social and non-social framings (r = 0.24, p = 0.23). Furthermore, when testing the correlation for each opponent's sophistication separately, we found that it was significant only in the control condition (r = 0.48, p = 0.0100, FWER_5%_  = 0.0102). Recall that *RB* is the only opponent, against which mentalizing should yield no advantage. Against other opponents, differences in performance induced by individual variability in attentional or motivational states are negligible, when compared to, e.g., differences induced by peoples' ToM sophistication. In brief, the inter-individual variability of peoples' performance against artificial mentalizing agents is unlikely to be driven by cognitive requirements (such as behavioural flexibility, working memory, inhibitory control, etc…) or attentional/motivational confounds. Rather, our analysis of peoples' earnings seems to indicate that peoples' ability to reliably predict the behaviour of artificial mentalizing agents critically depends upon whether or not they engage in (potentially automatic) sophisticated ToM inferences.

### Volterra decompositions

Next, we asked whether we could find evidence for framing-specific learning rules that could explain the observed differences in peoples' performances across framings. We thus performed Volterra decompositions of peoples' trial-by-trial choice sequences, i.e. we looked at how much trial-by-trial variance in peoples' choice sequences can be explained by the history of both players' actions.

Average Volterra's fit accuracy in each of the 4×2 conditions is given in Table 3 below. One can see that Volterra decompositions of participants' and artificial ToM agents' choices have similar fit accuracies. More precisely, they yield about 75% of correct choice predictions, which is significantly above chance level. This is a prerequisite for interpreting the estimated Volterra kernels as a summary of participants' average response to the history of players' actions.

**Table 3:** Average fit accuracy of Volterra decompositions of participants' choice sequences against each opponent (columns) in each framing condition (rows).

	RB	*0-ToM*	*1-ToM*	*2-ToM*
social framing	74.5%	76.5%	76.3%	76.2%
non-social framing	80.0%	82.2%	78.9%	79.6%

**Figure 4:**
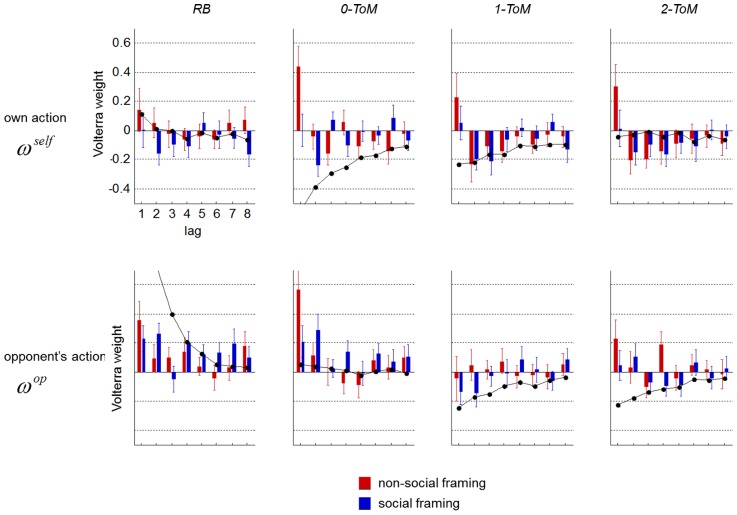
Volterra decomposition of participants' responses. Top: impulse response to participants' own action (x-axis: lag 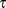, y-axis: Volterra weight 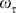) against each opponent (red: non-social framing, blue: social framing). Right: impulse response to participants' opponent's action. Errorbars depict one standard error on the mean. Black lines depict the "best *k-ToM* response" to each opponent type.

**Figure 5:**
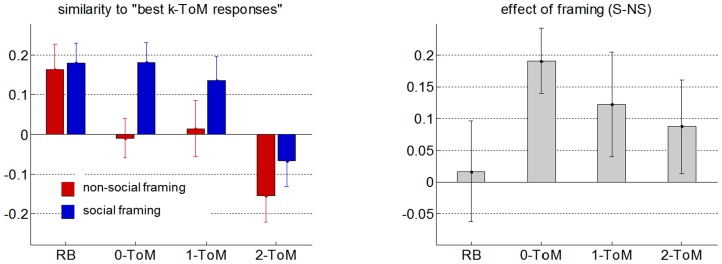
Optimality of participants' response. Left: group average correlation between participants' Volterra kernels and the "best *k-ToM* response" to each of the four different opponents (red: non-social framing, blue: social framing). Errorbars depict one standard error. Right: group average difference (social minus non-social) in the correlation between participants' Volterra kernels and the "best *k-ToM* response" to each of the four different.

Fig. 4 depicts the group mean Volterra kernels against each opponent, in the social and in the non-social framing condition. For each opponent, we superimposed the Volterra kernel of the corresponding "best *k-ToM* response", i.e. one ToM sophistication level above participants' opponents. For completeness, results of a parametric Volterra decomposition are exposed in Figure 5 of Text S1. In the non-social framing, it seems that people have a strong tendency to imitate their opponent's last action (cf. positive Volterra weight 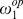). They also tend to perseverate, i.e. to reproduce their last choice (cf. positive Volterra weight 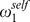). In the social condition, people rather seem to alternate their own actions (cf. negative Volterra kernels 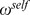) and to imitate their opponent's choices less often than in the non-social framing (cf. small Volterra kernels 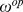). In addition, Volterra decompositions of peoples' choice sequences in the social framing seem much closer to the "best *k-ToM* response" than in the non-social framing (except maybe in the control condition).

First, we consider the impact of our experimental factors onto peoples' response to feedback history. The ANOVA on peoples' Volterra kernels confirms that both weights 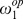 and 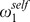 significantly decreased in the social framing, when compared to the non-social framing (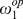: F = 6.6, p = 0.01; 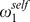: F = 13.7, p = 0.0003). Also, peoples' response to their opponent's past actions shows a main effect of opponent. More precisely, participants' tendency to replicate their opponents' actions decrease with the sophistication of their opponent (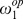: F = 11.5 p = 0.001, 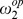: F = 6.8 p = 0.01). Note that there was no significant interaction between framing and opponent on Volterra weights (irrespective of the lag). This is interesting, because this means that our experimental factors have a similar effect on behavioural performance and on peoples' response to feedback history. Moreover, the observed change in Volterra kernels is consistent with the idea that peoples' effective ToM sophistication increases in the social framing, when compared to the non-social framing. This is because Volterra weights of mentalizing *k-ToM* agents are systematically smaller than those of *0-ToM* (cf. Fig. 1).

Next, we focus on the similarity to the "best *k-ToM* response", which we take as a proxy for the optimality of peoples' response. We measured the correlation between each participant's Volterra kernel and the appropriate "best *k-ToM* response" in each of the 4×2 conditions. This analysis is summarized on Fig. 5. One can see that the optimality score seems to mimic peoples' final earnings (cf. Fig. 3, bottom panels). In fact, people's optimality significantly correlated with their final earnings (r = 0.25, p = 0.0001), even after having removed the effect of the experimental factors (p = 0.002). We then performed an ANOVA on the Fisher-transformed correlation coefficients. Results showed that people's optimality significantly increased in the social framing, when compared to the non-social framing (F = 5.62, p = 0.02), and significantly decreased with the opponent's sophistication (F = 18.5, p = 0.0001). There was no significant interaction (F = 0.126, p = 0.723). Taken together, these results suggest that the effect of our experimental factors onto behavioural performance is mediated through peoples' similarity to the "best *k-ToM* response". A classical Sobel test [37] confirmed this for both framing (p = 0.010) and opponent (p = 0.013) factors.

In summary, our analysis of Volterra kernels demonstrates that the social framing induces a systematic change in peoples' behavioural response to feedback history. Importantly, this change is reminiscent of sophisticated meta-Bayesian inference, i.e. peoples' similarity to the "best *k-ToM* response" increases in the social framing, when compared to the non-social framing. This eventually drives peoples' behavioural performance against artificial mentalizing agents.

### Model inversions

**Figure 6:**
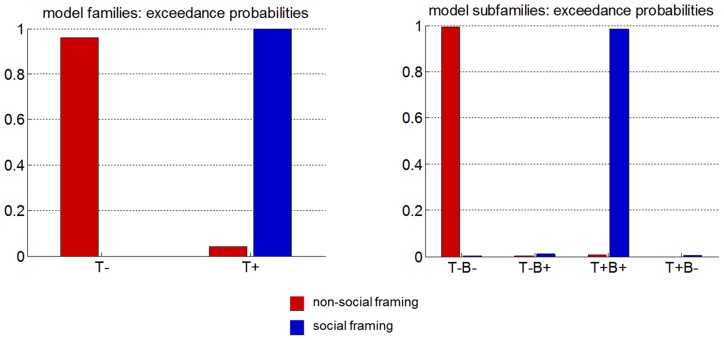
Bayesian model comparison. Left: exceedance probabilities of the no-ToM (T-) and ToM (T+) model families (red: non-social framing, blue: social framing). Right: exceedance probabilities of the no-ToM/non-Bayesian (T-B-), no-ToM/Bayesian (T-B+), ToM/bayesian (T+B+) and Tom/non-Bayesian (T+B-) model families.

Lastly, we performed a formal model-based analysis of peoples' trial-by-trial choice sequences, in the aim of identifying the most likely learning scenario in both social and non-social framings. In brief, we performed a group-level random-effect Bayesian model comparison (RFX-BMS, [36]) of fourteen different models (cf. Table 2). These include meta-Bayesian ToM models (*1-ToM*, *2-ToM* and *3-ToM*), non-Bayesian ToM models (*1-Inf* and *2-Inf*), Bayesian no-ToM models (*0-ToM*, *hBL*, *1-BSL*, *2-BSL* and *3-BSL*), as well as non-Bayesian no-ToM models (*RL*, *WSLS, Nash* and Volterra decompositions). In what follows, we will exploit these two orthogonal partitions of our model set, namely: T+/T- (which refers to models that include mentalizing or not) and B+/B- (which refers to models that rely upon Bayesian belief updates or not). Note that all models include a bias term that can capture a systematic tendency to prefer one alternative option over the other (within games/sessions). First, we performed Bayesian hypothesis tests to assess the stability of models attribution across conditions. To begin with, we tested the hypothesis that the model family (T+ versus T-) used in the social framing was the same than in the non-social framing, for each opponent. Evidence for the null hypothesis was found for the control condition *RB* (EP = 95%). However, evidence for a difference in model families across framings was found for both *0-ToM* (EP = 23%) and *1-ToM* (EP = 0%) opponents. The test was inconclusive for *2-ToM* (EP = 53%). Then, we tested whether the same family of model was used across opponents in a given framing. In this case, we found strong statistical evidence in favour of stability of model attributions. More precisely, the null hypothesis was strongly supported for all between-conditions comparisons (EP>83%), with the exception of comparisons between *2-ToM* and *RB* in the social framing, which yielded weaker evidence (EP = 69%). Overall, this analysis indicates that people's learning rule is mostly framing-dependent (but not opponent-dependent). This motivates our final analysis, which essentially is a framing-specific RFX-BMS. The result of this procedure is depicted on Fig. 6, which shows the exceedance probability of model families in both the social and non-social conditions. We refer the interested reader to Text S1 for quantitative diagnostics of the RFX-BMS approach (cf. fixed-effect analysis and confusion matrices).

One can see that, in the social condition, peoples' trial-by-trial choice sequences are more likely to be explained by T+ models than by T- models (EP = 100%). In contradistinction, peoples' behaviour in the non-social condition is more likely to be explained by models that do not rely on mentalizing (EP = 96%). This is strong statistical evidence that any realistic mechanistic description of peoples' policy in the social framing has to rely upon recursive mentalizing processes. We then asked whether we could find more specific evidence regarding the information-theoretic nature of peoples' belief updates. Thus, we further divided our T+ and T- families into B+ and B- subfamilies. We then used RFX-BMS to perform a comparison of the two corresponding subfamilies (T-B-, T-B+ in the non-social condition T+B+ and T+B+ in the social condition. We found that T+B+ models were the most likely explanations to peoples' trial-by-trial choices (EP = 98%) in the social condition, whereas T-B- was the most likely family in the non-social condition (EP = 99%). This is important, because this means that mentalizing processes are likely to follow meta-Bayesian belief update rules (as opposed to other non-optimal heuristics). In other terms, the way we learn about how others learn is near-optimal (from an information-theoretic point of view).

**Figure 7:**
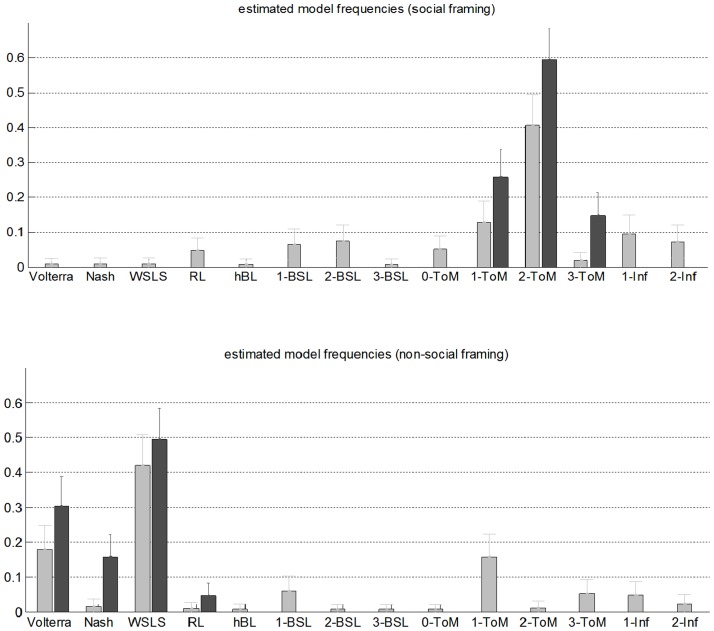
Distribution of ToM sophistication. Top: Estimated model frequencies in the social framing (dark grey: having restricted the models to the winning T+B+ family). Errorbars depict one posterior standard error. Bottom: Estimated model frequencies in the non-social framing (dark grey: having restricted the models to the winning T-B- family).

Let us now focus on the estimated models' frequency distribution in the social condition (cf. upper panel of Fig. 7). First, one can see that *2-ToM* is the most prevalent model (well above reference models such as *Nash* or *RL*). Second, we restricted the model comparison to the *T+B+* family, in the aim of deriving efficient estimates of the distribution of ToM sophistication in the human population. We found that *2-ToM* agents are about two times more frequent than *1-ToM* agents (*3-ToM* being almost negligible). This suggests that the natural inter-individual variability of ToM sophistication exists but is rather narrow. In addition, it is likely to be upper-bounded.

For completeness, Fig. 7 also shows the equivalent estimated models' frequency distribution in the non-social condition (cf. bottom panel). One may infer that *WSLS* is the most likely explanation for peoples' behaviour in this condition. However, it turns out that RFX-BMS may confuse Bayesian sequence learning with *WSLS* (more precisely: *2-BSL* or *3-BSL*). Although such statistical confusion does not compromise the interpretation of other potentially likely models, it renders the comparison of the families T-B- and T-B+ slightly unreliable. Thus, the estimation of model frequencies within the winning family (T-B-) is provided only as an indication (see Text S1 for further details).

## Discussion

Our study combined a computational modelling approach with an experimental investigation of Theory of Mind (ToM) in a situation of social interaction. We demonstrated a strong social framing effect, whereby the ability of participants to predict the behaviour of artificial mentalizing agents was conditional on whether or not they believed they were playing against another human being. Using data-driven analyses, we showed that this social framing effect was due to a difference in peoples' trial-by-trial response to feedback. In addition, we found that our meta-Bayesian model is a more plausible explanation of people's trial-by-trial choice sequences than other non-Bayesian and/or non-social (non-mentalizing) learning heuristics only in the social condition. Finally, we found statistical evidence that ToM sophistication is variable across people, and is likely to be upper-bounded (2-ToM).

Recall that our experiment aimed at revealing the specificity of social inference indirectly, by simulating behavioural data that conform to peoples' natural prediction of others' actions, and then measuring a difference in performance that originates from the task framing. Here, the framing induces priors that determine how people process the feedback information, which shapes their predictions regarding the next best move. Critically, such a manipulation only works if (i) the underlying model realistically simulates peoples' hidden social prior beliefs, and (ii) people are unlikely to appeal to these priors in the non-social framing. In our case, social priors essentially induce a sophisticated interpretation of the game's outcome, which involves mentalizing about others' beliefs. In turn, people engage in recursive belief updates, which we claim is very specific to human social interactions. To support this claim, we have provided two complementary pieces of evidence: (i) people could win over sophisticated (artificial) mentalizing agents only in the social framing condition, and (ii) the most likely explanation for people's trial-by-trial choices involves mentalizing only in the social condition. Note that the qualitative change in people's perspective induced by the framing is confirmed by the short debriefing we conducted at the end of the main experiment. In brief, most participants reported "having tried to adapt their strategy to their opponent's" in the social framing, whereas they were "looking for feedback temporal patterns" in the non-social framing. Some participants even reported that they perceived well that hiders were "responding to their own choices", whereas slot machines "followed complex, predetermined, sequences". Taken together, these results validate our meta-Bayesian model of mentalizing in repeated social interactions.

Perhaps the most shocking result of this work is the fact that people are clearly fooled by mentalizing (artificial) agents in the non-social condition. This happens despite repeated negative feedback that signals persistent prediction error. Note that this does not mean that people disregard this prediction error in the non-social condition; however, prediction error does not serve to learn the relevant variables. Our analyses suggest that the non-social framing of the task induces implicit priors that obscure the evidence for intentional behaviour. This is important, because this may explain why we engage in mentalizing as soon as we interact with social agents [1].

Note that one could argue that with sufficient training, participants would eventually learn the best response to their opponent, without having to mentalize. This is in principle possible, since *k*-ToM agents are reducible (up to 80% accuracy) to a linear convolution of competing players' actions (cf. Volterra decompositions in Fig. 1). However, there is hardly any sign of performance improvement over the entire session duration (cf. Figure 2 in Text S1).

A slightly more severe criticism of our interpretation of the social framing effect appeals to some form of systematic order effect between the social and the non-social conditions (the former was always performed after the latter). An example of this is [12], which shows that, e.g., pedagogical learning is facilitated when people are primarily engaged in teaching others. In our context, such order effect could not be driven by training or priming, which would rather improve peoples' performance in the non-social condition. In other words, our current (imbalanced) design could detect a net performance decline from the social to the non-social condition, above and beyond potential training and/or priming effects. Note that order effects could also be due to the impact of cognitive fatigue. Under the assumption that mentalizing is an effortful mental activity, one could argue that people may be less motivated to engage in sophisticated mentalizing in the second (non-social) condition, which would lead to performance losses. We will discuss motivational confounds below.

Even more problematic is the concern that the social framing effect might be confounded by some trivial difference in the understanding of the task structure (as induced by, e.g., peoples' assumptions regarding the way casino slot machines work). In particular, this implies that participants might have performed better in the non-social condition, had they been "warned" about the existence of some form of hidden sophisticated rule. Instead, we chose to favour a balanced design that relied on rather non-informative instructions. Critically however, participants' answers to our debriefing questions seem to indicate that they were well aware of the existence of some structure in the feedbacks' sequence (cf. above). Note that model comparisons of participants' trial-by-trial choices in the non-social framing yield ambiguous evidence either in favour of simple heuristics like "win-stay/lose-switch" or in favour of more sophisticated Bayesian sequence learning schemes (cf. confusion matrix in Figure 10 of Text S1). In addition, our analyses show that non-ToM sophisticated learning models do not seem to provide a likely explanation for peoples' trial-by-trial choices in the social condition. This means that sophisticated inferences induced by the social framing were specifically stemming from adopting the intentional stance [38], i.e. they assumed that the feedback sequence was the (potentially complex) result of their opponent's reaction to their own choices. Although this is certainly reassuring, we cannot entirely rule such potential confound out. We will address this potential design imbalance in forthcoming experiments.

Let us now briefly discuss potential attentional and/or motivational confounds. In brief, one could argue that the prospect of outsmarting some conspecifics (as opposed to some uninteresting machine) incites us to invest the mental effort required for performing sophisticated inferences (typically: mentalizing). In fact, our results rather speak against such attentional/motivational effects on peoples' performance (e.g., no framing effect against *RB*, no correlation between peoples' performance in the social and in the non-social framings…). In addition, we found no effect of framing on peoples' reaction times (see Text S1), which is surprising under such motivational interpretation (because one would expect people to respond faster in the social than in the non-social condition). In any case, such potential issues do not confound our main result, namely that one is unlikely to decipher intentional behaviour without *a priori* adopting the intentional stance [38].

Given the apparent added value of ToM sophistication, one might be surprised by its apparent limitation. In other words, one may wonder why evolution has not made all of us smarter. In fact, one can show that, in theory, competitive and cooperative social interactions induce both a lower and an upper bound on ToM sophistication [14]. Interestingly, the empirical estimate of the distribution of ToM sophistication levels (cf. bottom panel in Fig. 4) is very similar to the predicted equilibrium we derived from evolutionary game theory. Although this is certainly reassuring, it is yet unclear how such results would generalize over contexts that induce different incentives for sophisticated mentalizing. For example, the effort cost incurred when mentalizing in very complex settings might overcome the expected gain in performance. Thus, the cognitive process that yields the best complexity/accuracy trade-off might not involve ToM at all. This may explain why people tend to resort to rather heuristic behavioural policies in some complex social interactions. One can note however, that our upper bound on ToM sophistication (*2-ToM*) is consistent with results from behavioural economics regarding limited depth in strategic thinking. Experimental investigations of the cognitive hierarchy model, for instance, typically demonstrate that only a small proportion of people (around 20%) would exceed 2 steps of recursive thinking in strategic games (e.g., "beauty contest" games) [9]. Having said this, we would argue such strategic games are essentially different from our main task. This is because they monitor some form of explicit reasoning about others, whereas the time limitation on each trial of our main task rather reveals participants' intuitive "first guess" on their opponent (as is evident from peoples' short reaction times and the lack of effect of, e.g., working memory and inhibitory control on their performance in the main task). This relates to the current debate regarding the implicit/explicit dichotomy of mentalizing processes [39].

Let us now briefly discuss how novel or consistent our results are, when compared to to existing studies in both experimental psychology and behavioural economics. First, on the theoretical side, we bridged the gap between the literatures on strategic thinking in games [9], [10], [40], [41] and action understanding [11], [42], [43]. More precisely, we extended inverse planning models to situations of reciprocal social interactions, which may induce recursive beliefs. We also extended cognitive hierarchy models to repeated games, which may involve the (Bayesian) recognition of others' intentions and beliefs. The key point is that we can now mimic different sophistications of mentalizing. Second, on the experimental side, our results are consistent with the idea that learning in a social context relies on very specific cognitive processes, which are engaged for predicting others' behaviour (see, e.g., [22], [44]). In particular, previous neuroscientific studies have demonstrated that specific neural systems are activated when performing classical ToM tasks [45], [46] and during recursive thinking in games [22], [47]–[50]. In this context, our critical contribution was to demonstrate the added-value of (some form of) sophisticated mentalizing, in terms of its ability to decipher intentional behaviour. That is, we showed that, peoples' ability to predict goal-oriented choices critically depends upon whether they adopt the intentional stance [38] or not. This is not trivial, as one could think that domain-general learning heuristics could have performed well against mentalizing agents. Among the existing literature, the closest example to our work is [12], which shows that learners who know they are being explicitly taught (by a teacher) learn more from the data than when assuming otherwise. Taken together, our work and this recent study tend to contradict other existing studies that concluded that social learning (such as advice taking behaviour) was driven by non-specific reinforcement-like processes [44], [51]. Note however that no recursive learning models was considered for comparison purposes in these works.

Of course, our *k-ToM* model does not embrace all mentalizing processes. For example, it cannot be used to model how people "read others' mind" from low-level social signals such as eye gaze, bodily posture or facial expression [52]. Although it comprises the basic building blocks for modelling false beliefs (cf. beliefs about beliefs), it would still require some modification to capture the difference between people who pass and people who fail the false belief test [53] (but see [54]). We note that extending *k-ToM* in order to explain the various phenomena observed across the literature is well beyond the scope of the present study. We will pursue this in subsequent publications.

Finally, we would like to highlight a few promising applications of this work. Given the simplicity of the task that participants have to perform (namely: choosing between two alternative options, one of which is leading to a reward), one could argue that it could be used to address three aspects of mentalizing. First, one could assess its developmental aspect by quantifying the drift in ToM sophistication that occurs when we age. Second, our approach could be adapted to perform ethological inter-species comparisons of ToM sophistication (e.g. monkeys, great apes and humans). Third, in line with ideas from the emerging field of computational psychiatry [55], [56], one may wish to quantify pathological impairments of mentalizing in neuropsychiatric disorders such as autism or schizophrenia. We are currently pursuing these ideas. In these contexts, the main added-value of our approach lies in its ability to capture quantitative differences in ToM sophistication through its impact on behaviour, without being confounded by linguistic skills.

## Supporting Information

Text S1 This is a document containing supporting information regarding models, statistical methods, experimental details, additional data analyses and model inversion diagnostics.
(PDF)
