# Supplementary material for: The Social Bayesian Brain: Does Mentalizing Make a Difference When We Learn?
Source: PLoS Comput Biol. 2014 Dec 4;10(12):e1003992. doi: 10.1371/journal.pcbi.1003992 (PMC4256068; doi:10.1371/journal.pcbi.1003992)
Supplement: Text S1 — This is a document containing supporting information regarding models, statistical methods, experimental details, additional data analyses and model inversion diagnostics. (PDF) [file pcbi.1003992.s001.pdf]

The social Bayesian brain:

does mentalizing make a difference when we learn?

Supporting information

M. Devaine[1], G. Hollard[2], J. Daunizeau[1,3]

[1] Brain and Spine Institute, Paris, France

[2] Maison des Sciences Economiques, Paris, France

[3] ETH, Zurich, France

Address for correspondence:

Jean Daunizeau

Motivation, Brain and Behaviour Group

Brain and Spine Institute

47, bvd de l'Hopital, 75013, Paris, France.

Tel: +33 1 57 27 43 26

Fax: +33 1 57 27 47 94

Mail: jean.daunizeau@gmail.com

Materials and methods

Deriving the Bayesian update rule of 0-ToM

In the following, we will posit that *0-ToM* observers *a priori* believe that the probability of her opponent's choice may vary smoothly over time (as in a –bounded- random walk). For numerical reasons, the corresponding prior transition density is defined on log-odds x_t^0 rather than on probabilities themselves, i.e.:

$$\begin{aligned} p\left(x_{t+1}^0 \middle| x_t^0\right) &= N\left(x_t^0, \sigma^0\right) \\ p_t^{op} &\equiv s\left(x_t^0\right) \end{aligned} \quad \text{(A1)}$$

where $N(a,b)$ denotes the Gaussian probability density function with mean a and variance b, $s: x \to 1/1+\exp(-x)$ is the sigmoid mapping, and σ^0 is the prior volatility of *0-ToM*'s opponent log-odds x_t^0. Note that the volatility σ^0 is known to *0-ToM* and does not have to be learned. This is in fact the main difference between *0-ToM* and the model *HGF*, which includes an update rule for σ^0 (Mathys et al. 2011). We assume that *0-ToM* learns by assimilating new observations recursively in time or trials, as follows:

$$\begin{aligned} p\left(x_{t+1}^0 \middle| a_{1:t}^{op}\right) &= \int q\left(x_t^0\right) p\left(x_{t+1}^0 \middle| x_t^0\right) dx_t^0 \\ q\left(x_{t+1}^0\right) &\propto p\left(a_{t+1}^{op} \middle| x_{t+1}^0\right) p\left(x_{t+1}^0 \middle| a_{1:t}^{op}\right) \end{aligned} \quad \text{(A2)}$$

where $q\left(x_{t+1}^0\right) \equiv p\left(x_{t+1}^0 \middle| a_{1:t+1}^{op}\right)$ is *0-ToM*'s posterior belief about the log-odds x_{t+1}^0, conditional upon observed actions a^{op} up to trial $t+1$. Equation A2 is a Bayes-optimal probabilistic scheme for online tracking of the log-odds, whose first line is *0-ToM*'s prediction about her opponent's

behavioural tendency, whereas its second line derives from Bayes' rule (see, e.g., Daunizeau et al., 2009). Let us assume that *0-ToM* holds a Gaussian probabilistic belief $q\left(x_t^0\right)=N\left(\mu_t^0,\Sigma_t^0\right)$ about the log-odds at trial t, where μ_t^0 and Σ_t^0 are the sufficient statistics of q (i.e. its first- and second-order moments). This implies that her prediction is Gaussian as well, with inflated second-order moment (cf. Equation A1), i.e.: $p\left(x_{t+1}^0 \middle| a_{1:t}^0\right)=N\left(\mu_t^0,\Sigma_t^0+\sigma^0\right)$. This yields the following expression for the next (log-) posterior density $L\left(x_{t+1}^0\right)\equiv\log q\left(x_{t+1}^0\right)$ (Daunizeau et al. 2010):

$$L\left(x_{t+1}^0\right)=-\frac{1}{2}\frac{1}{\Sigma_t^0+x_1^0}\left(x_{t+1}^0-\mu_t^0\right)^2+\log s\left(x_{t+1}^0\right)+\left(a_{t+1}^{op}-1\right)x_{t+1}^0+cst \quad \text{(A3)}$$

where the constant is the log-normalization factor. The first iteration of the Laplace approximation (Friston et al., 2007) consists in approximating L by its second-order Taylor expansion around μ_t^0, and deriving the approximate first- and second-order moments of the corresponding Gaussian density from there on, as follows:

$$\begin{aligned} L\left(x_{t+1}^0\right) &\approx L\left(\mu_t^0\right)+L'\left(\mu_t^0\right)\left(x_{t+1}^0-\mu_t^0\right)+\frac{1}{2}L''\left(\mu_t^0\right)\left(x_{t+1}^0-\mu_t^0\right)^2 \\ \Rightarrow q\left(x_{t+1}^0\right) &\approx N\left(\mu_{t+1}^0,\Sigma_{t+1}^0\right):\begin{cases}\mu_{t+1}^0=\mu_t^0-L''\left(\mu_t^0\right)^{-1}L'\left(\mu_t^0\right)\\ \Sigma_{t+1}^0=-L''\left(\mu_t^0\right)^{-1}\end{cases}\end{aligned} \quad \text{(A4)}$$

where the derivatives of L are evaluated at μ_t^0:

$$\begin{aligned} L'\left(\mu_t^0\right) &= a_{t+1}^{op}-s\left(\mu_t^0\right) \\ L''\left(\mu_t^0\right) &= -\frac{1}{\Sigma_t^0+\sigma^0}-s'\left(\mu_t^0\right) \\ s'\left(\mu_t^0\right) &= s\left(\mu_t^0\right)\left(1-s\left(\mu_t^0\right)\right)\end{aligned} \quad \text{(A5)}$$

The limitations of such "early-stopping" variant of the Laplace approximation are discussed in Mathys et al., (2011). Inserting Equation A5 into Equation A4 yields *0-ToM*'s learning rule:

$$\begin{aligned} \mu_{t+1}^0 &= \mu_t^0 + \Sigma_{t+1}^0 \left(a_{t+1}^{op} - s\left(\mu_t^0\right) \right) \\ \Sigma_{t+1}^0 &= \left(\frac{1}{\Sigma_t^0 + \sigma^0} + s'\left(\mu_t^0\right) \right)^{-1} \end{aligned} \quad \text{(A6)}$$

where the right-hand term is the explicit form of the evolution function of the sufficient statistics of *0-ToM*'s belief about the log-odds x^0.

Finally, *0-ToM*'s prediction $\hat{p}_t^{op}$ about her opponents' move at trial t is the expected sigmoid mapping of the log-odds x_t^0, having observed his opponent's behaviour a^{op} up to trial $t-1$:

$$\begin{aligned} \hat{p}_t^{op} &= E\left[p_t^{op} \middle| a_{1:t-1}^{op} \right] \\ &= E\left[s\left(x_t^0\right) \middle| a_{1:t-1}^{op} \right] \\ &\approx s\left(\mu_t^0 \Big/ \sqrt{1 + \left(\Sigma_{t-1}^0 + \sigma^0\right) 3/\pi^2} \right) \end{aligned} \quad \text{(A7)}$$

where the third line derives from a moment-matching approximation to the logistic density (see Daunizeau 2014). Note that $\hat{p}^{op}$ is a sigmoidal function of μ and becomes non-informative when the posterior uncertainty Σ grows to infinity (i.e.: $p^{op} \xrightarrow{\Sigma \to \infty} 1/2$). This concludes the derivation of Equation 3 of the main text.

Volterra decompositions of choice sequences

Volterra series allow a systematic decomposition of dynamical systems' input-output relationships, where the output is typically a function of the history of past inputs. In our context, this means fitting the following logistic convolution model:

$$p\left(a^{self} \middle| \omega\right) = \prod_t q_t\left(\omega\right)^{a_t^{self}} \left(1 - q_t\left(\omega\right)\right)^{1-a_t^{self}}$$
$$q_t\left(\omega\right) = s\left(\omega^0 + \sum_\tau \omega_\tau^{op}\left(2a_{t-\tau}^{op} - 1\right) + \sum_\tau \omega_\tau^{self}\left(2a_{t-\tau}^{op} - 1\right)\right) \quad \text{(A8)}$$

where $q_t\left(\omega\right) = p\left(a_t^{self} = 1 \middle| \omega\right)$ is the probability that the agent choses the first option at trial t and τ is some arbitrary time lag. In Equation A8, ω^0 is a bias term that captures a potential average tendency to favour one of the alternative options. Here, the choice of the input basis functions (a^{op} and a^{self}) was motivated by:

- their simplicity;
- their completeness, i.e. a^{op} and a^{self} is the actual available information at each trial, (e.g., the game's outcome can be derived as a nonlinear function of both players' actions);
- the fact that they induce a very efficient Volterra decomposition of reinforcement learning algorithms.

First-order Volterra kernels ω^{op} (resp. ω^{self}) capture the impact of lagged opponent's (resp. own) actions a^{op} (resp. a^{self}) onto peoples' choice probability. First-order Volterra kernels would be equivalent to impulse response functions, would the system be linear. Note that this analysis is essentially similar to Lau & Glimcher (2005).

We estimated each participant's Volterra kernels ω^{op} and ω^{self} in each condition of the 2x4 factorial design. This was done using a variational Bayesian approach (Daunizeau 2014), which outputs both posterior estimates and the model evidence. The latter allows Volterra decompositions to enter group-level Bayesian model comparison (see main text). In this context, it serves as a reference for agents' models.

In total, there are $2\tau+1$ free parameters in Equation 8 (one Volterra kernel per input basis function, plus the bias term). In the main text, we present Volterra decompositions with a maximum lag of $\tau_{\max}=8$. This means that we estimated 15 parameters from a sequence of 60 binary choices. To regularize the estimation, we also performed parametric Volterra decompositions. These consist in constraining the Volterra kernels ω_τ to be exponential functions of the lag, i.e.: $\omega(\tau)=\omega_0\exp(-\gamma\,\tau)$, where ω_0 and γ control the amplitude and the decay rate of the Volterra kernel, respectively. Results of this analysis are given below.

RFX-BMS: Group-level Bayesian model selection

All models m have unknown parameters θ, whose impact on the data y is nonlinear and obscured by measurement noise. This is why we rely upon variational approaches to approximate Bayesian inference (Beal 2003), which regularize model fit using shrinkage priors on model parameters. More precisely, the VBA-toolbox uses a variational Bayesian scheme that recovers both the approximate posterior density $q(\theta)\approx p(\theta|y,m)$ and a free energy bound F to the log model evidence $\log p(y|m)$ under the Laplace approximation (Daunizeau 2014), given participants' choice sequences:

$$
\begin{aligned}
F &= I(\mu) + \frac{1}{2}\ln|\Sigma| + \frac{p}{2}\ln 2\pi \\
\mu &= \arg\max_{\theta} I(\theta) \\
\Sigma &= -\left[\left.\frac{\partial^2 I}{\partial\theta^2}\right|_{\mu}\right]^{-1} \\
I(\theta) &= \ln p(y|\theta,m) + \ln p(\theta|m)
\end{aligned}
\qquad \text{(A9)}
$$

where $I(\theta)$ is the log joint density over data y and parameters θ under the generative model m, p is the number of parameters, and μ and Σ are the mean and variance of the approximate Gaussian posterior density $q(\theta) = N(\mu,\Sigma)$. Equation A9 can be considered a pseudo-code for VBA's model inversion. Shrinkage priors $p(\theta|m) = N(0,1)$ on model parameters were i.i.d. normal with moments set to 0 (mean) and 1 (variance). Note that varying the priors did not change the nature of the results. The likelihood term $p(y|\theta,m)$ was defined according to the softmax policy in Equation 1 of the main manuscript, where the influence of parameters θ depends upon the model m.

Equation A9 grand-fathers heuristic penalized likelihood scores for model comparison, such as BIC or AIC (Penny 2012). In our context, it was used to approximate 14X26X2X4=2912 model evidences (14 models, 26 participants, 2 task framings, 4 opponents), given each participant's choice sequence in each condition of the main task. These summary statistics were then taken to a random-effect group-level Bayesian model selection (RFX-BMS), as follows.

RFX-BMS assumes that the population is composed of subjects that differ in terms of the model that describes them best. In this view, an experiment is a poll that randomly samples n subjects from the population, who are labelled according to their corresponding model. Let r_k be the

frequency of models of type $k = 1,...,K$ in the population (where K is the total number of models), and m_i be the ith subject's label, where $i = 1,...,n$. The probability of observing any given label m_i is determined by the respective frequency r_k of each model and has the following multinomial distribution:

$$p(m_i|r) = \prod_{k=1}^{K} r_k^{m_{ik}}$$
$$m_{ik} = \begin{cases} 1 & \text{if } k = l \\ 0 & \text{otherwise} \end{cases} \tag{A10}$$

where m_i is a one-in-K label vector, i.e. the index l of the non-zero entry encodes the subject's model. Given a group of n labelled subjects, one can interrogate the posterior density $p(r|m)$ on the unknown model frequencies in the population. But of course, one typically does not know what the subjects' labels are. Instead, labels m are observed indirectly, through subject-level model log-evidences $L_{ik} = \log p(y_i|m_{ik} = 1)$, which encode how likely the ith subject's dataset y_i is under the kth model. This induces a hierarchical probabilistic model that can be inverted using either sampling (e.g. Gibbs) or variational approaches, to derive the posterior density $p(r|y)$ over model frequencies, given subjects' data (Stephan 2009):

$$p(r|y) \propto p(r) \sum_{m} \left[\prod_{i=1}^{n} p(y_i|m_i)\, p(m_i|r) \right]$$
$$p(y_i|m_i) = \exp\left(\sum_{k=1}^{K} m_{ik} L_{ik} \right) \tag{A11}$$

where $p(m_i|r)$ is given in Equation A10 and the prior $p(r)$ is typically set to a non-informative (flat) density, i.e.: $p(r) \propto 1$. It follows that differences in model evidences L_{ik} will be expressed

in the posterior distribution $p(r|y)$, which will deviate from the prior, i.e.: $E[r_k|y] > 1/K$ for some models.

One can also derive the so-called *exceedance probability* (EP) φ_k – the probability that the kth model is more frequent in the population than any other models (given observed data): $\varphi_k = P(r_k \geq r_{k' \neq k}|y)$. As with model frequencies, the EPs satisfy: $0 \leq \varphi_k \leq 1$ and $1 = \sum_{k=1}^{K} \varphi_k$. For example, when comparing two models, EPs verify: $\varphi_1 = P(r_1 \geq 1/2|y) = 1 - \varphi_2$. In general, EPs express a degree of (posterior) confidence on the difference between model frequencies.

Finally, it may turn out that no model clearly dominates the model comparison (e.g., $\varphi_k \approx 1/K$ for all models). One can then resort to family inference, which amounts to partitioning the model set into $v = 1,...,V$ subsets (model families). The subset f_v contains all models belonging to the vth family. The family frequencies s are therefore given by: $s_v = \sum_{m \in f_v} r_v$, and one can derive family EPs: $\varphi_v = P(r_v \geq r_{v' \neq v}|y)$.

Note that this idea is used to assess the stability of models across conditions, which we call “between-conditions” RFX-BMS (Rigoux 2013). One can think of two conditions as inducing an augmented model space composed of K^2 2-tuples that encode all combinations of candidate models and conditions. Here, any 2-tuple identifies the models associated with each condition (which may or may not be the same), and its log-evidence is derived by summing up the corresponding log model evidences over conditions. To assess the probability that the same model underlies both conditions, one uses family inference on a partition of the K^2 tuples that divides them into a first subset, in which the same model underlies both conditions, and a

second subset containing the remaining tuples (with distinct condition-specific models). The ensuing family EP then measure the probability that different conditions most frequently correspond to different models.

Details about the experimental procedure

The experiment was run at the Laboratoire d'Economie Expérimentale de Paris (LEEP, Paris Experimental Economics Laboratory). We performed two experimental sessions on two different days with two different groups of people. Recruitment of participants was performed through the data base of the LEEP.

Participants of each group were welcomed together in the same room, and a computer was randomly attributed to each participant. Small separations between participants' computers prevented them to communicate or look at other participants' screen during the experiment.

Before the beginning of the experiment, people were instructed that they could not communicate with each other, that they could freely call off the experiment at any point and that they would receive a monetary bonus that would depend on their performance in the different tasks. Each of the different tasks was then briefly described, along with its payment rule (see below). At this point, participants were invited to ask any question regarding the experiment.

Once the set-up was clear for all participants, the experimental session started. At the beginning of each task, written instructions were displayed on each participant's computer screen.

At the end of the experiment, participant came individually into the "control cabin" of the room to receive payment and answer a few debriefing questions. Participants were first asked to describe their strategy during both the hide and seek and casino games. Then, they were asked to report any perceived differences between the different players and sessions. Finally, they

were invited to freely comment on their subjective experience during these two games, as well as during the other tasks.

Below are the payment rules for each task, in the order they were presented and ran by participants:

- *Hide and Seek*: you will play 4 games of Hide and Seek against 4 different players. At the end of the experiment one of the four games will be randomly selected, and each correct answer will yield .15€.
- *Vicky's Violin task* : This task is not financially rewarded.
- *MCST*: this task is composed of 40 trials, and each correct answer will yield .05€.
- *Casino Task*: you will play 4 sessions of the game. At the end of the experiment one of the four games will be randomly selected, and each correct answer will yield .15€.
- *Frith-Happé animations*: this task is composed of 20 trials, and each correct answer will yield .10€.
- *Go-No Go Task*: you will be rewarded according to the number of errors (false alarm or missed trials) you make. For instance making less than 3 errors will yields 4€ whereas making more than 40 errors will lead to no monetary payoff.
- *Empathy Quotient*: This task is not financially rewarded..
- *3-back task*: this task is composed of 400 trials, and each correct answer will yield .02€.
- *Imposing Memory Task*: this task is composed of 40 trials, and each correct answer will yield .07€.

Results

In what follows, actions a^{op} and a^{self} take binary values encoding the first ($a=1$) and the second ($a=0$) available options, by convention. The game outcome $u_t = U\left(a_t^{self}, a_t^{op}\right)$ at trial t can take the value 1 ("correct") or 0 ("incorrect"). Peoples' performance in each condition of the main task is defined as the total difference between the numbers of correct and incorrect trials, i.e. $\sum_t \left(2u_t - 1\right)$.

Note: although we report summary statistics that are not corrected for multiple comparisons, we indicate the family-wise error rate threshold (*FWER$_{5\%}$*) when necessary. More precisely, we used the standard Sidak correction, i.e.: $FWER_{5\%} = 1 - 0.95^n$, where n is the number of multiple tests (Sidak 1967).

Design sanity check

Although the *k-ToM* algorithms were developed without any systematic preference for a given alternative action, their behavioural policy is stochastic in nature. This could have resulted in non-negligible biases that could be different across framing conditions. In turn, this would induce a confound in our interpretation of the pattern of participants' performances across conditions. Thus, we performed the following analysis. First, we measured the absolute bias $\bar{b}$ of each opponent, against each participant, in each condition:

$$\bar{b} = \left| \frac{1}{T} \sum_{t=1}^{T} a_t^{op} - \frac{1}{2} \right| \qquad \text{(A12)}$$

By construction, a "fair coin" (chance level of 50%) would have zero absolute bias. Figure 1 below depicts the average absolute bias for each opponent in each framing.

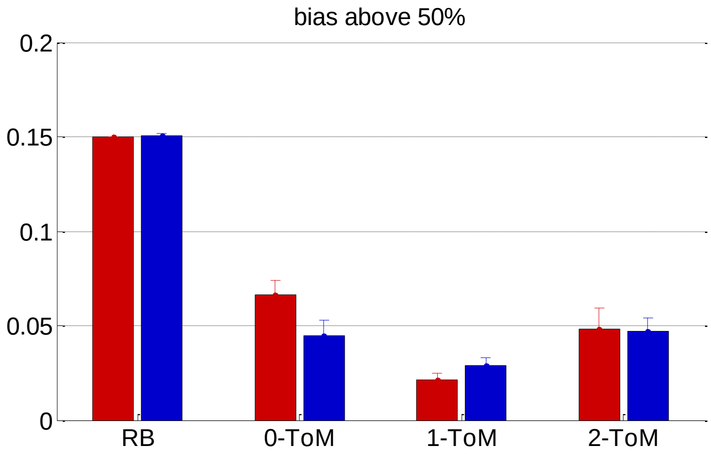

Figure 1: average opponents' bias. Group average absolute biases of the four different opponents, plus or minus one standard error (red: non-social framing, blue: social framing).

Reassuringly, *RB* exhibits a bias at exactly 65%. One can also see that, on average, *k-ToM* algorithms are left with a small bias of about 55%. We then performed an ANOVA to assess the effect of framing, opponent and their interaction on the opponents' biases. Results show an effect of opponent ($F=155.1$, $p<10^{-5}$), but no effect of framing ($F=0.7$, $p=0.40$) or interaction ($F=2.1$, $p=0.11$). This is important, because this makes the small residual bias in the *0-ToM*, *1-ToM* and *2-ToM* conditions an unlikely explanation for peoples' performance pattern. In particular, the residual bias cannot explain the observed performance difference between framings.

Dynamics of condition-specific earnings in the main task

Figure 2 below shows the dynamics of cumulated earnings across trials.

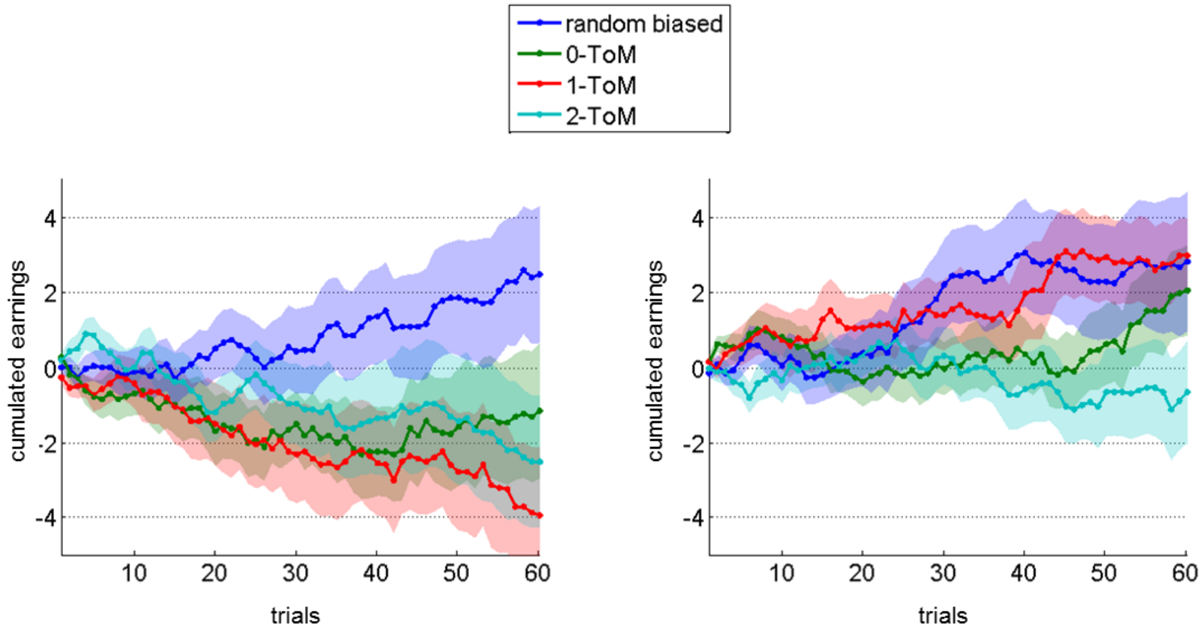

Figure 2: Participants' performance dynamics. Left: group-mean cumulated earnings (y-axis) as a function of trials (x-axis), against each opponent in the non-social framing condition (blue: random biased, green: 0-ToM, red: 1-ToM, magenta: 2-ToM). Shaded areas depict one standard error. Right: same format, social framing.

Based on final earnings only, we had summarized the results as follows: In the non-social framing, participants seem to continuously lose against all mentalizing opponents, be even with *0-ToM*, and win against *RB*. In the social framing, participants seem to win against all artificial agents except *2-ToM* (null earnings). It is reassuring to see that overall, visual extrapolations of accumulated earnings yield qualitatively similar predictions.

Reaction times analysis

Participants' reaction time was recorded on each trial of each condition of the main task. Figure 3 below summarizes the results in terms of mean reaction times (in log space).

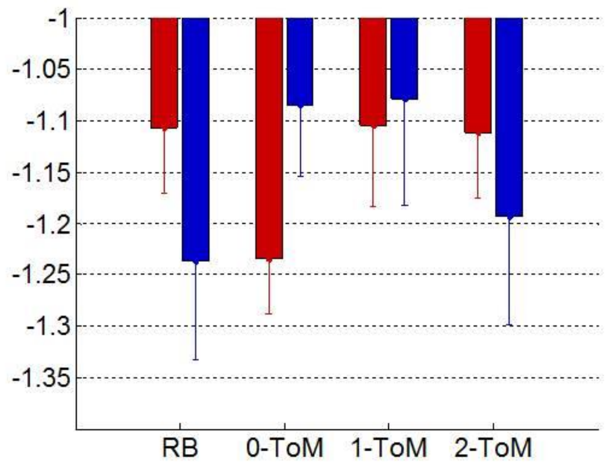

Figure 3: average participants' log- reaction times. Group average log- reaction times against the four different opponents, plus or minus one standard error (red: non-social framing, blue: social framing).

One can see that there is none of our experimental factors (opponent type and task framing) appears to have a clear impact on peoples' reaction times. In fact, this is confirmed by an ANOVA, which shows no evidence for a main effect of framing (p=0.80) or of opponent type (p=0.33).

Effect of performance in the secondary tasks

We analysed the impact of the performances in the seven secondary tasks onto peoples' performance in each session of the main task using a general linear model, which also included

participants' age and gender. We used omnibus F-tests to test for the effect of any of the secondary tasks on peoples' performance in the main task. First, no effect was found in the social (F=0.63, p=0.72) or in the non-social (F=1.55, p=0.38) conditions, when final earnings were averaged across opponents. This holds true for the difference between the social and non-social framings (F=2.13, p=0.10).

Table 1 below gives the F scores and p-values of the condition-specific omnibus test on the impact of executive functions/empathy/ToM tasks onto peoples' performance in each of the 4X2 conditions.

	random	0-ToM	1-ToM	2-ToM
social	F=1.12<br>p=0.39	F=1.04<br>p=0.44	F=0.79<br>p=0.61	F=0.59<br>p=0.75
non-social	F=0.912<br>p=0.52	F=0.94<br>p=0.49	F=2.44<br>p=0.06	F=2.28<br>p=0.07

Table 1: F statistics and p-values for the omnibus effect of executive functions, empathy and ToM task on each 2X4 conditions of the main task (columns: four opponents, rows: two task framings).

One can see that none of these tests reaches the 5% false positive rate significance threshold. Only when we looked at the opponent-specific difference in accumulated earnings between framings did we find an effect (omnibus F-test: F=2.65, p=0.04). More precisely, participants' performance against 1-ToM increases with their performance in the Frith-Happé task (t=2.3, p=0.02), but is not significantly related to other tasks. This makes sense, given that performance in this task is related to the ability to discriminate between intentional and physical causation. Figure 4 bellow summarizes the respective impact of executive functions/empathy/ToM tasks

onto the difference in peoples' performance between the social and non-social framings, against 1-ToM.

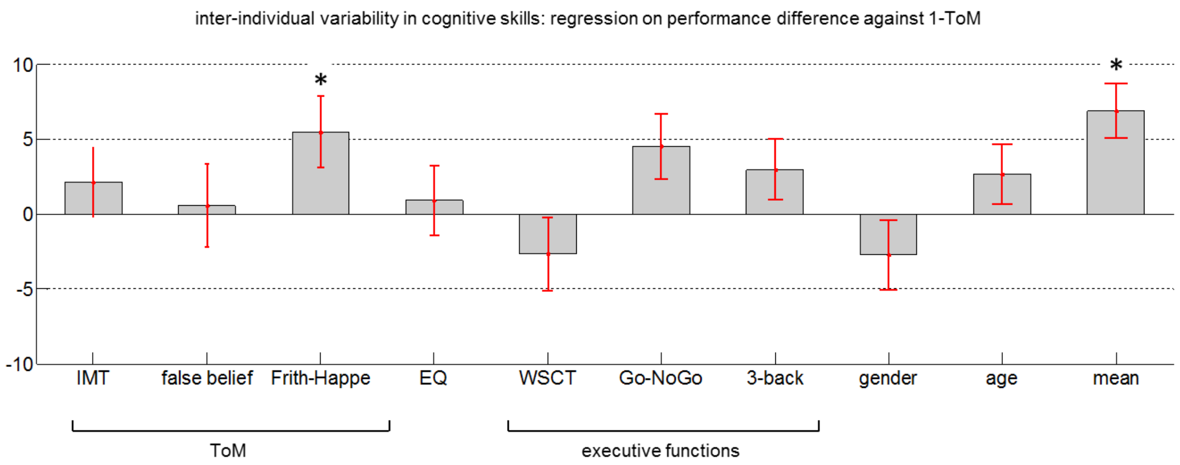

Figure 4: Effect of the performance in the secondary tasks on the difference in cumulated earnings between the social and the non-social condition against 1-ToM. Estimated GLM regression coefficients, plus or minus one standard error (red: non-social framing, blue: social framing).

Note however that this result is but a statistical tendency, since the corresponding statistical tests were not corrected for multiple comparisons ($FWER_{5\%}$=0.0064).

Parametric Volterra analyses

Figure 5 below summarizes the result of parametric Volterra decompositions of participants' choices sequences (using exponential Volterra kernels, see above paragraph).

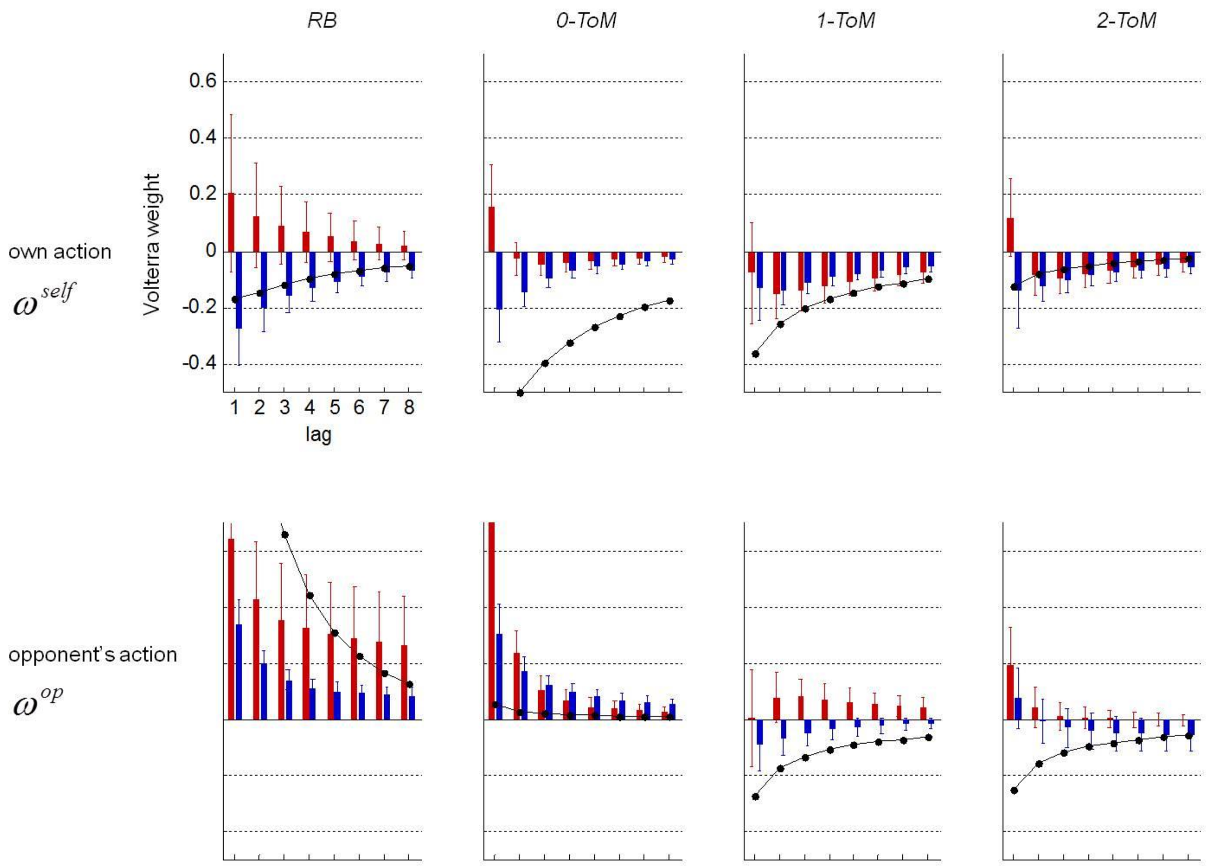

Figure 5: Parametric Volterra decompositions of participants' responses. This figure uses the same format as Fig. 4 in the main manuscript (blue=social framing, red=non-social framing).

It is reassuring to see that the results of the parametric and non-parametric Volterra analyses are qualitatively similar to each other. However, the main effects of our experimental factors (framing and opponent type) are somewhat easier to eyeball in the parametric setting.

RFX-BMS diagnostics

In complement to random-effect Bayesian Model Selection (RFX-BMS), we derived simple group-level summary statistics of model inversions.

Figure 6 below depicts the group-mean log evidence of each model (summed over opponents), for both framings.

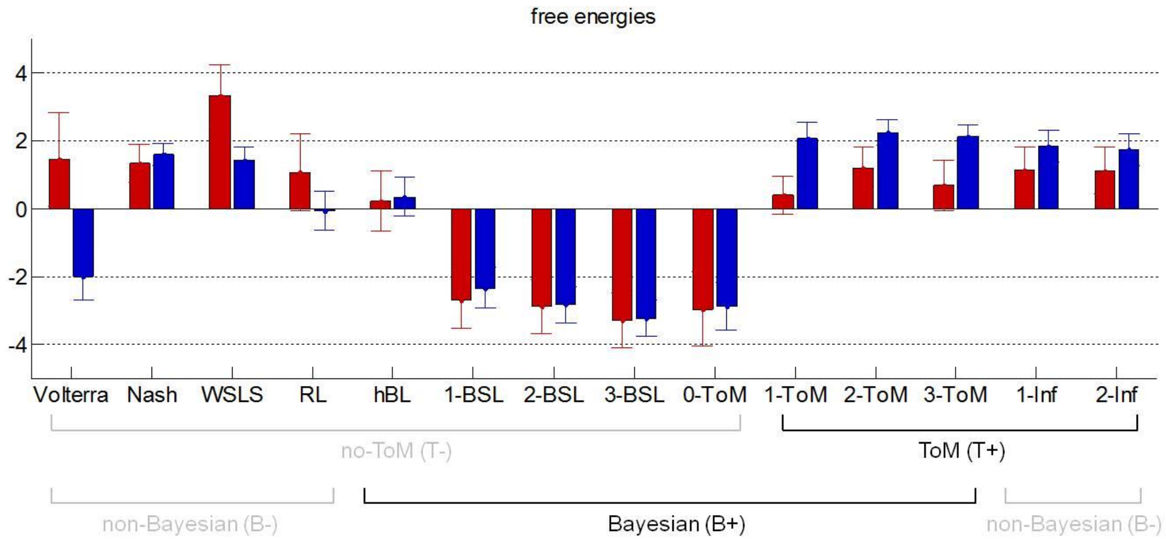

Figure 6: Average log model evidences. Errorbars depict one standard error (blue=social framing, red=non-social framing). Models are partitioned into the ToM/no-ToM families, as well as Bayesian/non-Bayesian families.

Note that log-evidences in Fig. 6 have been mean-corrected. Recall that no direct comparison between log-evidences in different framings is possible (e.g., one cannot compare the likelihood of a given model in the social versus the non-social framing).

In the social framing, although no model clearly stands out as being more probable than others, one can see that T+ models dominate. In the non-social framing however, it seems that the WSLS strategy is the likeliest explanation for participants' trial-by-trial responses.

Results of the RFX-BMS demonstrate that most participants behave as a *2-ToM* agent in the social framing (i.e. *2*-ToM has the maximum model frequency, cf. Fig. 7 in the main manuscript). However, there is a strong variability in *2-ToM*'s fit accuracy across subjects, which explains why

2-ToM does not clearly single out on Fig. 6. This is illustrated on Figure 7 below, which shows *2-ToM*'s fit quality for both the best and the worst subject in the social framing (across opponent types).

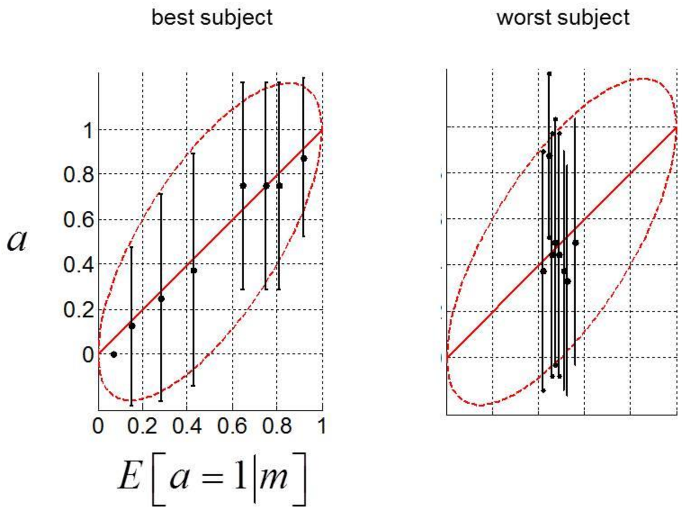

Figure 7: Best and worst subjects' fits. Participant's observed choice data (y-axis) as a function of *2-ToM*'s fitted data (x-axis). Right: *2-ToM*'s best fit. Left: *2-ToM*'s worst fit. The model's fitted output is the expected choice data $E\left[a_t = 1 \middle| m\right]$ at each trial t, marginalized over model parameters. Here, the series of expected choice data was binned into eight quantiles, each of which corresponds to a subset of observed choice data with a given mean (black dots) and standard deviation (black errorbars). An ideal model fit would align along the plain red line, with errorbars matching the dashed red ellipse.

One can see that *2-ToM*'s fit quality varies from almost perfect fit (left panel of Fig. 7), to clearly poor fit accuracy (right panel of Fig. 7). This simply indicates that *2-ToM* may not be the best explanation for all subjects. In other words, it is likely that the population is composed of subjects that differ in terms of the model that describes them best (cf. main assumption of RFX-BMS).

Between-condition RFX-BMS

Figure 8 below summarizes our between-condition RFX-BMS, performed for each experimental factor (framing and opponent type) separately. The main objective of this analysis is to address the question of whether our experimental factors induced a difference in model family (T+ or T-) or not. When assessing the impact of the framing factor, we report the exceedance probability (EP) that peoples' behaviour in the social and in the non-social framing most frequently correspond to the same family, for each opponent type. When assessing the impact of the opponent factor, we report the EP that peoples' behaviour against two different opponents most frequently correspond to the same family, for each framing.

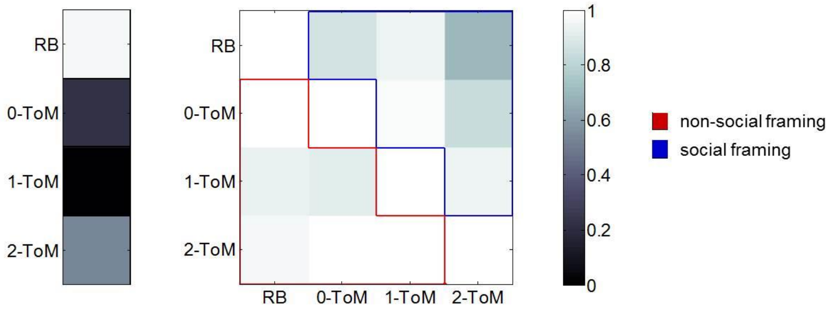

Figure 8: between-condition RFX-BMS. Impact of the experimental factors (left: framing, right: opponent type) in terms of EPs. Note that matrix elements on the right panel are framing-specific (blue upper-right triangle: social framing, red lower-left triangle: non-social framing).

A small EP indicates that peoples' behaviour in the corresponding pair of conditions is likely to be best described by different model families. One can see that the opponent type factor has a much smaller impact on the best description of peoples' behaviour than the framing factor. This

is confirmed by eyeballing condition-specific RFX-BMS analyses, which are summarized in Figure 9.

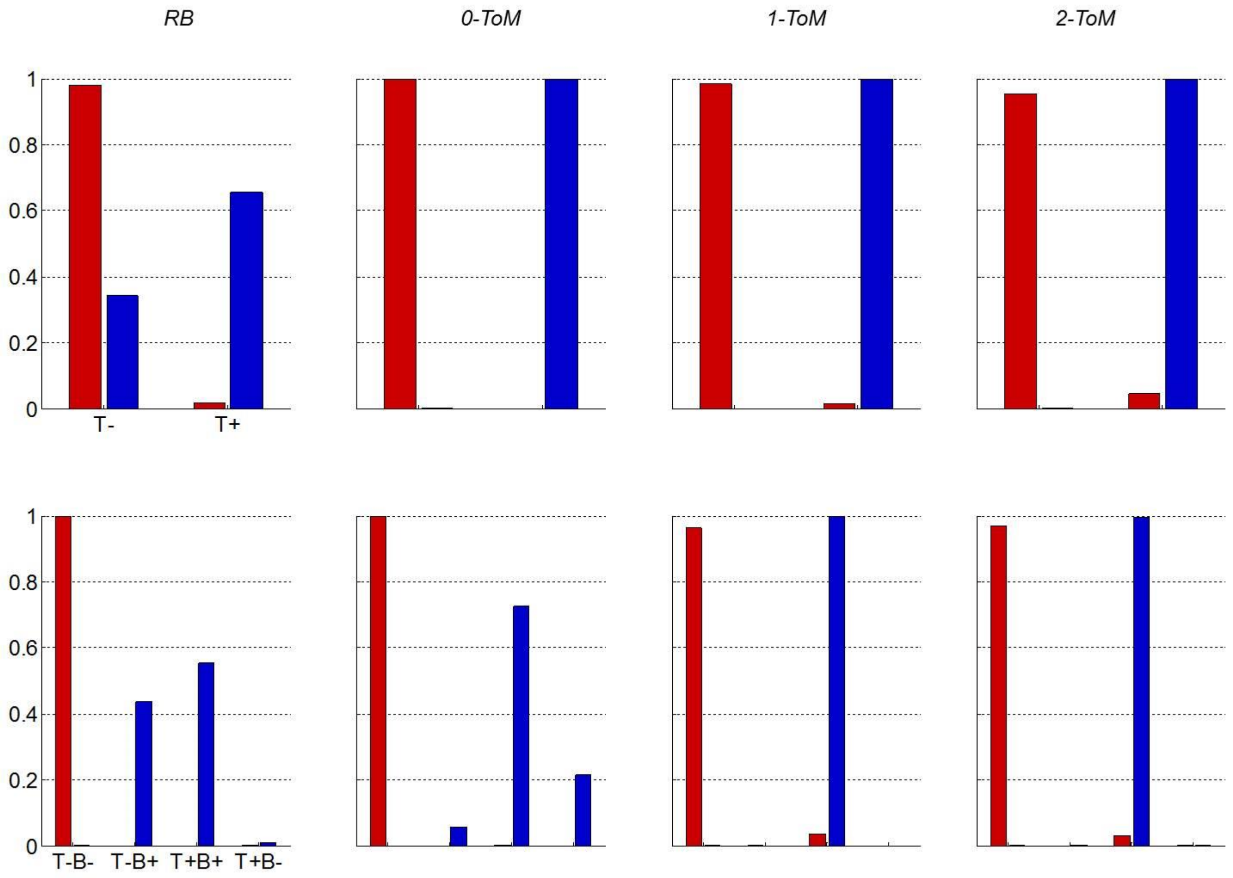

Figure 9: Condition-specific RFX-BMS. This figure uses the same format as Fig. 6 of main text. Top: EP of both T+ and T- model families for each opponent type (from left to right: *RB*, *0-ToM, 1-ToM* and *2-ToM*) and each framing (blue: social framing, red: non-social framing). Bottom: EP of T-B-, T-B+, T+B+ and T+B- model subfamilies for each opponent type and each framing.

RFX-BMS: model identifiability

Different models may yield similar predicted choice sequences, which may confuse Bayesian model selection. We thus performed Monte-Carlo simulations designed to quantify model identifiability, under conditions similar to our experimental data analyses.

We first generated choice sequences under each agent's model (13 models, 60 trials per game, 4 opponents, 26 dummy subjects). For each simulated data, we performed a Bayesian Model Selection, based upon the VB approximation to the log evidence of each of the 13 candidate models. For any given type of simulated data, we then measured the frequency with which each candidate model is eventually selected. The so-called *confusion matrix* derives from renormalizing these frequency profiles, to yield the probability of having simulated the data under each model, given that a particular candidate model was selected. It is shown on Figure 10 below. Any non-diagonal element in this matrix signals a potential confusion between the inferred model and the true (hidden) model. More precisely, the ith row shows how often each model was actually generating the data, given that the ith model was identified as the most likely.

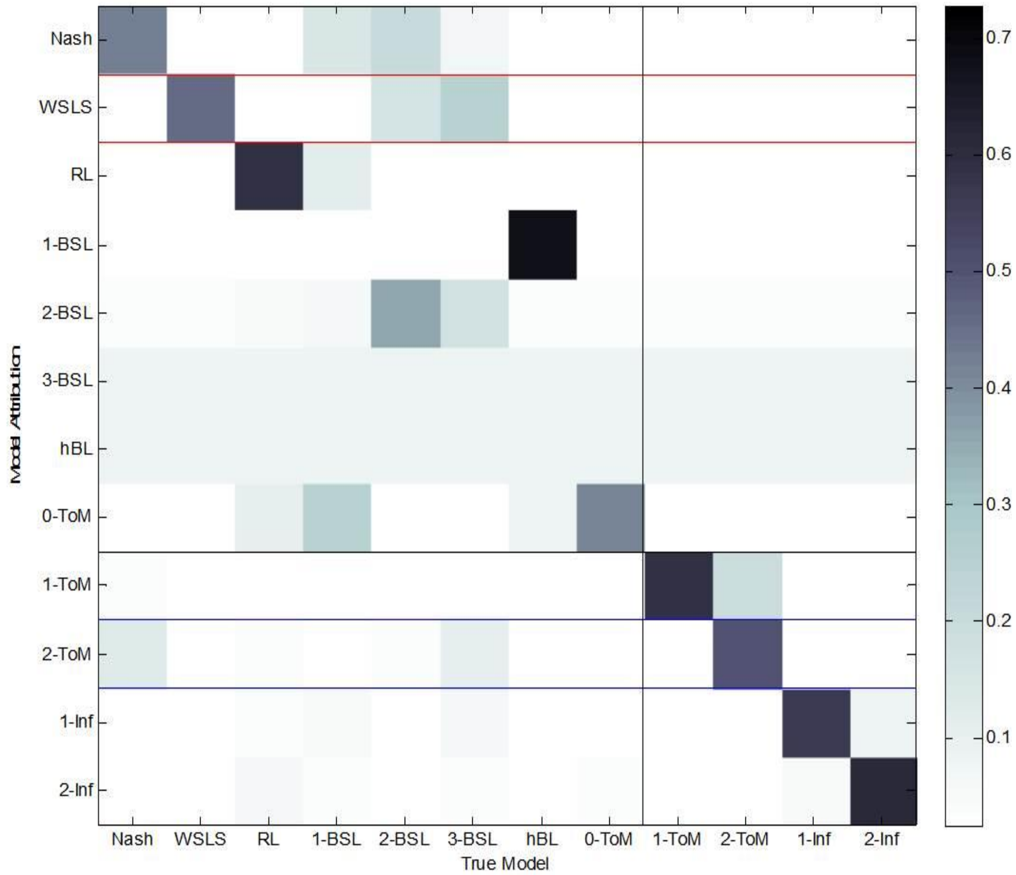

Figure 10: confusion matrix. Probability of the true model (x-axis), given each model attribution (y-axis). Perfect model identifiability would exhibit no extra-diagonal non-zero element. Black lines indicate the T+/T- model partition. Coloured lines highlight the confusion profiles of the most likely models in each framing (blue: social framing, red: non-social framing).

First of all, one can see that there is almost possible confusion between models belonging to the T+ family, and models belonging to the T- family. In addition, there is almost no confusion between models within the T+ family (lower-right quadrant). However, there are partial model non-identifiabilities within the T- family (upper-left quadrant). In particular, eventually selecting the model *1-BSL* is in fact strong evidence for data generated under the model *hBL*. To a much

lesser extent, eventually selecting the model WSLS may in fact be taken as evidence for Bayesian sequence learning (*2-BSL* and *3-BSL*). This is important, since *WSLS* is the most likely model in the non-social framing (cf. Fig. 7 in the main text).

References

Beal M. (2003), *Variational algorithms for approximate Bayesian inference*, PhD thesis, ION, UCL, UK.

Daunizeau J., Friston K.J., Kiebel S.J. (2009), *Variational Bayesian identification and prediction of stochastic nonlinear dynamic causal models*. Physica D: nonlinear phenomena, 238: 2089-2118.

Daunizeau J., Den Ouden H. E. M., Pessiglione M., Stephan K. E., Kiebel S. J., Friston K. J. (2010), *Observing the observer (I): meta-Bayesian models of learning and decision-making*. PLoS ONE, 5(12): e15554.

Daunizeau J. (2014), *On the exponential, sigmoid and softmax mappings*. Document available at: http://sites.google.com/site/jeandaunizeauswebsite/links/resources.

Daunizeau J, Adam V, Rigoux L (2014) *VBA: a probabilistic treatment of nonlinear models for neurobiological and behavioural data*. PLoS Computational Biology 10: e1003441.

Friston K, Mattout J, Trujillo-Barreto N, Ashburner J, Penny W (2007) *Variational free energy and the Laplace approximation*. NeuroImage 34: 220–234.

Lau B., Glimcher P. W. (2005), *Dynamic response-by-response models of matching behavior in rhesus monkeys*. J. Exp. Ana. Behav. 84(3): 555-79.

Mathys C., Daunizeau J., Friston K., Stephan K. (2011), *A Bayesian foundation for learning under uncertainty*. Frontiers Hum. Neurosci., 5: 39.

Penny W. D. (2012). *Comparing dynamic causal models using AIC, BIC and free energy*. NeuroImage 59(1), 319-30.

Rigoux L, Stephan KE, Friston KJ, Daunizeau J (2013) *Bayesian model selection for group studies - Revisited*. NeuroImage 84C: 971–985.

Sidak Z. K. (1967), *Regular confidence regions for the means of multivariate normal distributions*. J. Am. Stat. Assoc. 62 (318): 626-633.

Stephan KE, Penny WD, Daunizeau J, Moran RJ, Friston KJ (2009) *Bayesian model selection for group studies*. NeuroImage 46: 1004–1017.
